# Naringin Exerts Therapeutic Effects on Mice Colitis: A Study Based on Transcriptomics Combined With Functional Experiments

**DOI:** 10.3389/fphar.2021.729414

**Published:** 2021-08-24

**Authors:** Jianyi Dong, Yuanyuan Chen, Fang Yang, Weidong Zhang, Kun Wei, Yongjian Xiong, Liang Wang, Zijuan Zhou, Changyi Li, Jingyu Wang, Dapeng Chen

**Affiliations:** ^1^ Comparative Medicine Department of Researching and Teaching, Dalian Medical University, Dalian, China; ^2^ Central Laboratory, First Affiliated Hospital of Dalian Medical University, Dalian, China; ^3^ Labarotary Animal Center, Dalian Medical University, Dalian, China

**Keywords:** naringin, inflammatory bowel disease, RNA sequencing, peroxisome proliferator-activated receptor-γ, NF-κB

## Abstract

Naringin has been shown to exert protective effects in an animal model of ulcerative colitis, but detailed mechanisms remain unclear. This study aimed to investigate function and signaling mechanisms underlying naringin-induced therapeutic effects on colitis. Two mouse models were established to mimic human Inflammatory bowel disease (IBD) by treating drinking water with dextran sodium sulphate or intra-colonic administration of 2, 4, 6-trinitrobenzene sulfonic acid. Transcriptomics combined with functional experiments were used to investigate underlying mechanisms. Colitis symptoms, including weight loss and high disease activity index were significantly reversed by naringin. The inflammatory response, oxidative reactions, and epithelial cell apoptosis that occur with colitis were also alleviated by naringin. After naringin treatment, transcriptomics results identified 753 differentially expressed mRNAs that were enriched in signaling pathways, including the neuroactive ligand-receptor interaction, calcium signaling, and peroxisome proliferator-activated receptor (PPAR) signaling. The naringin-induced alleviation of colitis was significantly inhibited by the PPAR-γ inhibitor BADGE. In IEC-6 and RAW264.7 cells incubated with lipopolysaccharide (LPS), NF-κB-p65, a downstream protein of PPAR-γ, was significantly increased. Naringin suppressed LPS-induced high expression of NF-κB-p65, which was inhibited by small interfering RNA targeting PPAR-γ. Our study clarifies detailed mechanisms underlying naringin-induced therapeutic effects on mice colitis, and PPAR-γ was found to be the main target of naringin by functional experiments both *in vivo* and *in vitro*. Our study supplies new scientific information for the use of naringin in colitis treatment.

## Introduction

Inflammatory bowel disease (IBD) is an array of chronic inflammatory disorders within the gastrointestinal tract and includes primarily ulcerative colitis (UC) and Crohn’s disease (CD). CD affects the entire gastrointestinal tract from mouth to anus, whereas UC mainly affects colon (Sairenji et al., 2017). Since 2000, incidence ranges of CD are 6–11/100,000 and that of UC are 6–15/100,000 in the Western world (Kaplan and Windsor, 2021). The main symptoms of IBD include diarrhea, abdominal pain, mucus stool and bloody stool, which can be accompanied by a variety of intestinal and parenteral complications, such as intestinal obstruction, stenosis, fibrosis, and joint lesions. Moderate and severe UC or colonic CD is a high-risk factor for colon cancer (Grivennikov, 2013). IBD has become one of the most complex and refractory intestinal diseases which bring a heavy economic and social burden to the health systems of various countries all over the world.

The IBD pathogenesis is still under investigation, recent studies have suggested that both genetic and environmental factors are involved. Current drugs include sulfasalazine, 5-aminosalicylic acid, broad spectrum antibiotics, and corticosteroids are often applied in the treatment of IBD (Abraham et al., 2017). However, many side effects like nausea, anorexia, cytopenia, myalgia, and malfunctions of the kidney, liver, and lungs have been reported (Abraham et al., 2017). Although biopharmaceuticals have been shown to exert good therapeutic effects on IBD, a large part of patients do not respond or lose response to biopharmaceuticals over time (Yanai and Hanauer, 2011). In addition, biologic therapy may increase the risk of serious infections and malignancies (Holmer and Singh, 2019). Therefore, it is valuable to screen or develop new drugs to expand therapy options.

In recent years, herbal medicines with multi-target actions have been widely used in the treatment of complex inflammatory diseases. Citrus peel is a kind of herbal medicine and is used as an anti-inflammatory drug in China. Naringin (4′,5,7-trihydroxyflavanone-7-rhamnoglucoside) is a major and active flavanone glycoside isolated from citrus fruit species (Ali and El Kader, 2004). Naringin has been found to exert anti-inflammatory, anti-oxidative, and anti-cancer effects (Jeon et al., 2001; Zhang et al., 2016). Cao et al. find that naringin exerts protective effects on dextran sodium sulphate (DSS)-induced colitis *via* modulation of PPAR-γ activity (Cao et al., 2018). However, the detailed mechanisms of naringin on colitis are still not fully understood because multiple targets may be involved. In addition, naringin-induced modulation of PPAR-γ should also be confirmed in cells and other animal models of colitis.

In this study, transcriptomics and functional experiments are used to uncover the detailed mechanisms of naringin-induced therapeutic effects on colitis. RNA sequencing (RNA-seq) is a deep-sequencing approach in transcriptome profiling that provides an impartial and accurate method for measuring the levels of transcripts and their isoforms (Kukurba and Montgomery, 2015). Two mouse models are established to mimic human IBD by treating drinking water with DSS or intra-colonic administration of 2, 4, 6-trinitrobenzene sulfonic acid (TNBS).

## Materials and Methods

### Materials

Naringin (purity ≥ 98%) was purchased from Beijing Solarbio Co. Ltd. (Beijing, China). Sulfasalazine (SASP) was purchased from Tian-jin Kingyork Group Co. Ltd. (Tianjin, China). Antibodies against PPAR-γ (A0270) were from ABclonal Co. Ltd. (Wuhan, China). Antibodies against caspase-3 (19677-1-AP), cleaved caspase3 (cl-caspase3) (19677-1-AP), and iNOS (14142-1-AP) were from Proteintech Co. Ltd. (Wuhan, China). Antibodies against NF-κB-p65 (66535-1-Ig), phosphorylated NF-κB-p65 (p-NF-κB-p65) (WL02169) and PPAR-α (WL00978) were from Wanleibio Co. Ltd. (Shenyang, China). A cell counting kit-8 (WLA074b) was from Wanleibio Co. Ltd. BADGE (B6691) was obtained from Apexbio (Houston, TX, United States). Chemicals were obtained from Sigma–Aldrich (St. Louis, MO, United States), unless otherwise indicated.

### Animals

Eighty male C57BL/6 mice (6–8 weeks old, weighing 18–20 g) were obtained from the laboratory animal center, Dalian Medical University, Dalian city, China [Certificate of Conformity: No. SYXK (Liao) 2018–0007]. The experimental protocol was approved by Dalian Medical University Animal Care and Ethics Committee (No. AEE20046). The animals were acclimatized to laboratory conditions (23°C, 12 h/12 h light/dark, 50% humidity, ad libitum access to food, and water) for 2 weeks prior to experiments. The animal protocol was designed to minimize pain and discomfort to the animals. Mice were housed one per cage and were deprived of food for 12 h before experiments. All mice were euthanized by barbiturate overdose (intravenous injection, 150 mg/kg pentobarbital sodium) for intestinal tissue collection. All animal experiments were carried out in accordance with the National Institutes of Health guide for the care and use of laboratory animals. Animal studies are reported in compliance with the ARRIVE guidelines (Kilkenny et al., 2010). All applicable institutional and/or national guidelines for the care and use of animals were followed.

### Cell Culture and Cell Transfection

Murine RAW264.7 cells and rat intestinal IEC-6 epithelial cells were purchased from the cell bank of the Shanghai Institute (Shanghai, China). Cells used in this study were evaluated before conducting experiments, and no significant interspecies variations in PPAR-γ signaling that might have affected the results of the current study were observed in preliminary tests. The cells were maintained at 37°C in a 5% CO_2_ environment. The RAW264.7 cells were cultured in RPMI-1640 medium with 10% top fetal bovine serum. Rat intestinal IEC-6 epithelial cells were cultured in DMEM (Invitrogen, Waltham, MA, United States) medium with 4.5 mg/ml glucose, 50 U/mL penicillin, 50 U/mL streptomycin, 4 mM glutamine, 25 mM HEPES, and 10% fetal bovine serum (Invitrogen) (Sui et al., 2020). RAW264.7 and IEC-6 cells were transfected using Lipofectamine 2000 (Invitrogen) with PPAR-γ-targeted or control small interfering RNA (siRNA) oligos (GenePharma, Suzhou, China), according to the manufacturer’s instructions (Takara Biotechnology (Dalian) Co., Ltd.). The siRNA sequences for PPAR-γ interfering RAW264.7 cells and IEC-6 cells, respectively were:

Sense: 5′-GGU​GCU​AAG​AGA​UUG​CCU​UTT-3′;
Antisense: 5′-AAG​GCA​AUC​UCU​UAG​CAC​CTT-3′;
Sense: 5′-CCA​UCC​GAU​UGA​AGC​UUA​UTT-3′;
Antisense: 5′-AUA​AGC​UUC​AAU​CGG​AUG​GTT-3′.
The efficiency of gene silencing was confirmed by western blotting.

### Experimental Design

For the DSS–colitis model, mice were divided randomly into five groups of six mice each. The mice were treated as follows: group I was a sham-operated control with gavage administration of saline; group II was untreated colitis; and groups III, IV, and V were treated with the following: SASP (500 mg/kg body weight, gavage administration, dissolved in saline), low-dose naringin (20 mg/kg body weight, gavage administration, dissolved in saline), and high-dose naringin (40 mg/kg body weight, gavage administration, dissolved in saline), respectively. All treatments occurred 1 day after colitis induction with DSS. SASP is an anti-inflammatory drug that is, widely used for the clinical treatment of diseases such as IBD and, therefore, it was used as a positive control for the effects of naringin on colitis. Mice in group III, IV, and V were administered SASP or naringin by gavage once a day for seven successive days. Mice in groups II–V were given drinking water containing DSS (4% w/v) dissolved in autoclaved distilled water, to induce colitis symptoms (Feng et al., 2020). Control group I mice were given autoclaved distilled water and otherwise, treated in the same way. No abnormal moribund mice were found during the study. Food intake and bodyweight of mice were recorded once a day. On the 8th day, mice in group I–V were given autoclaved distilled water as drinking water. On the 9th day, distal colon samples in groups I–V were harvested for biochemical studies. Mice in the PPAR-γ inhibition group were injected intraperitoneally with 30 mg/kg BADGE solution per day for seven consecutive days, the experimental designs are the same as above.

For the TNBS–colitis model study, mice were divided randomly into five groups of six mice each. The mice were treated as follows: group I was a sham-operated control with gavage administration of saline; group II was untreated colitis; and groups III, IV, and V were treated with the following: SASP (500 mg/kg body weight, gavage administration, dissolved in saline), low-dose naringin (20 mg/kg body weight, gavage administration, dissolved in saline), and high-dose naringin (40 mg/kg body weight, gavage administration, dissolved in saline), respectively. All treatments occurred 1 day after colitis induction. Mice in group III, IV, and V were administered SASP (Xiong et al., 2017), naringin by gavage once a day for seven successive days. Colitis was induced with TNBS, as described previously (Dong et al., 2020). A catheter was inserted through the anus to approximately the level of the splenic flexure under urethane anesthesia. The colon was then infused with 0.1 ml of TNBS dissolved in ethanol (50% v/v) at a dose of 125 mg/kg. The mice could eat and drink ad libitum starting 1 h after the operation. On the 8th day, mice in groups I–V were given autoclaved distilled water as drinking water. On the 9th day, distal colon samples in groups I–V were harvested for biochemical studies. Protein extraction, western blotting, and ELISA were performed as previously described (Dong et al., 2020).

### Assessment of Disease Activity Index

Mice body weights were monitored daily. The DAI scores were assessed according to our previous study (Melgar et al., 2005). Briefly, an observer blinded to the treatments combined the scores for weight loss, stool bleeding, and stool consistency on the last day.

### Isolation of Colonic Epithelial Cells

Colonic epithelial cells were isolated according to a previous study (Dong et al., 2020). Briefly, distal colons were isolated from euthanized mice and immediately rinsed with ice-cold phosphate-buffered saline (PBS) to clear luminal contents. The distal colon was then opened longitudinally along the mesenteric border. Tissue was cut into approximately 2 mm long pieces and submerged in 40 ml of ice-cold PBS with 5 mM EDTA in a 50 ml Falcon tube. The pieces of tissue in PBS-EDTA were then incubated at 37°C with gentle rocking for 30 min. Following incubation, colonic tissue was vigorously shaken to disperse colonic crypts and surface epithelium in solution. Supernatant was then loaded into 1.5 ml microcentrifuge tubes and spun at 1 × 10^3^ g for 5 min to pellet suspended cells. The isolated epithelial cells were used in downstream applications.

### RNA-Seq and Bioinformatics

Three naringin treated DSS-induced C57BL/6 mice, three DSS-induced C57BL/6 mice and three untreated mice were used for mRNA profile detection. Total RNA was extracted using TRIzol reagent (Invitrogen), and quality was evaluated using the RNA Nano 6000 Assay Kit of the Bioanalyzer 2,100 system (Agilent Technologies, CA, United States).

A total of 3 µg RNA per sample was used to generate an mRNA sequencing library using NEBNext^®^ Ultra™ RNA Library Prep Kit for Illumina^®^ (NEB, United States) following manufacturer’s recommendations and index codes were added to each sample for identification (Gao et al., 2012). Briefly, mRNA was purified and fragmented. Then, first and second strand cDNA was synthesized, ends were repaired and adenylated, adapters were ligated, and fragments were enriched with PCR amplification. Library quality was assessed on the Agilent Bioanalyzer 2,100 system. Libraries were sequenced on an Illumina platform.

Raw data (raw reads) of fastqc format were first processed through Trimmomatic (Bolger et al., 2014). In this step, clean data (clean reads) were obtained by removing reads containing adapter, reads containing poly-N, and low-quality reads from raw data. Reference genome and gene model annotation files were downloaded from the genome website directly. Index of the reference genome was built and paired-end clean reads were aligned to the reference genome using Hisat2 (v2.0.5), the selected mapping tool. The mapped reads of each sample were assembled by StringTie (v1.3.3b) in a reference-based approach. FeatureCounts v1.5.0-p3 was used to count the reads mapped to each gene. Then, FPKM of each gene was calculated based on the length of the gene and read counts mapped to this gene (Conesa et al., 2016). FPKM, expected number of Fragments Per Kilobase of transcript sequence per Million base pairs sequenced, considers the effect of sequencing depth and gene length for the read counts at the same time. Differential expression analysis of two conditions/groups (two biological replicates per condition) was performed using the DESeq2 R package (1.16.1). The resulting *p*-values were adjusted using the Benjamini and Hochberg approach. Genes with an adjusted *p*-value < 0.05 found by DESeq2 were assigned as differentially expressed. We used the clusterProfiler R package to test the statistical enrichment of differential expression genes in KEGG pathways. The raw sequencing data sets were deposited in the Sequence Read Archive of the NCBI (https://www.ncbi.nlm. nih.gov/sra) under accession number PRJNA741857.

### Quantitative Real-Time Polymerase Chain Reaction

Validation of differentially expressed genes was performed by qRT-PCR using SuperReal PreMix Plus (SYBR Green, FP205, Tiangen) with an Applied Biosystems StepOnePlus Real-Time PCR System, according to the manufacturer’s instructions. Total RNA was isolated from colonic epithelial cells using RNAiso Plus (9108, TaKaRa) and reverse-transcribed to single-stranded cDNA using a reverse transcription system (KR116, Tiangen). Primer sequences are listed in Table 1. A separate qRT-PCR using a primer for the detection of GAPDH was used as a control.

**TABLE 1:** mRNA sequences obtained from the NCBI database.

Gene name	Forward primer sequence (5’−>3′)	Reverse primer sequence (5’−>3′)<
Mus-Ppar-α	tatggccgagaagacgcttg	att​ctg​tga​gct​ccg​tga​cg
Mus-Ppar-γ	atccaagacaacctgctgca	caa​gga​gga​cag​cat​cgt​ga
Mus-Ppar-δ	gcccaagttcgagtttgctg	acc​tgg​ggc​aca​ttc​atg​ag
Mus-Scd1	ttctcagaaacacacgccga	tcc​agt​ttt​ccg​ccc​ttc​tc
Mus-Hmgcs2	gcccaaacgtctagactccc	ttg​ctt​gtg​tct​cca​ggt​gg

### Hematoxylin-Eosin, Immunohistochemical and Immunofluorescent Analysis

Colon tissues were fixed with 4% paraformaldehyde at 4°C. The samples were dehydrated, embedded in paraffin, and sectioned into 3-µm-thick transverse sections. For H and E staining, the sections were dewaxed, rehydrated, and stained with hematoxylin and eosin. After being washed with distilled water and dehydrated, the sections were treated with xylene. Colitis activity scores were assessed in a blinded fashion, by combining the scores of inflammation severity, inflammation extent, crypt damage, and percent involvement (Kihara et al., 2003). Inflammation severity score was assessed according to the following criteria: 0: none; 1: mild; 2: moderate; and 3: severe. Inflammation extent score was assessed according to the following: 0: none; 1: mucosa; 2: submucosa; and 3: transmural. Crypt damage score was assessed by the following criteria: 0: none; 1: basal 1/3 damage; 2: basal 2/3 damage; 3: crypt lost; surface epithelium present; and 4: crypt and surface epithelium lost. Percent involvement score was determined as follows: 0: 0%; 1: 1–25%; 2: 26–50%; 3: 51–75%; and 4: 76–100%. For IHC staining, the sections were dewaxed, rehydrated, and washed with distilled water three times. The sections were then treated with 3% H_2_O_2_ for 10 min, washed in PBS containing Tween-20 (PBST), treated with 5% bovine serum albumin (BSA) (Solarbio, A8020) and then incubated with rabbit anti-PPAR-γ (ABclonal, A0270) (1:100 dilution) overnight at 4°C. After three washes with PBST, the sections were incubated with secondary antibody for 30 min at 37°C. After being rinsed, the sections were incubated with diaminobenzidine. After counterstaining with hematoxylin, washing with distilled water, and dehydration, the sections were treated with xylene. For IF staining, sections were dewaxed, dehydrated, and subjected to antigen retrieval. 3% BSA (Solarbio, A8020) was utilized to block nonspecific binding. Then, the sections were incubated with rabbit anti-PPAR-γ (ABclonal, A0270) (1:100 dilution) overnight at 4°C. Later, these sections were rinsed with PBS and incubated with Alexa Fluor 488 (FITC) secondary antibody (Proteintech, SA00013-2) in the dark for 60 min at room temperature. 4,6-diamidino-2-phenylindole (Invitrogen, P36941) was later applied to dye the nuclei. Fluorescence photographs were obtained under a fluorescence microscope DM5000 B (Leica, Germany).

### Statistical Analysis

The animal experiments, *in vitro* experiments, and data analysis were conducted according to a single-blind study design. Data were compared between three or more groups using one-way ANOVA, and between two groups using Student’s t-test. Data were expressed as the mean ± standard deviation. Data were normally distributed, and each group showed similar variances. Further evaluation was carried out using Kruskal–Wallis rank sum tests. All experiments were repeated at least three times and a *p* value < 0.05 was considered statistically significant.

## Results and Discussion

### Effects of Naringin on Normal Mice and Cells

**FIGURE 1:**
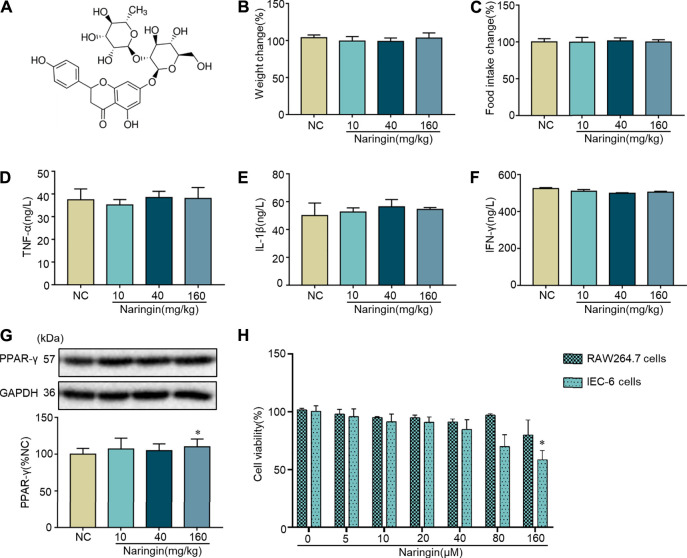
Toxicity of naringin in normal cells and mice. **(A)** Chemical structure of naringin. Naringin was administered to mice by gavage once a day for seven consecutive days. Effects of naringin on **(B)** body weight and **(C)** food intake. Expression levels of the colonic cytokines **(D)** TNF-α, **(E)** IL-1β, and **(F)** INF-γ were examined by ELISA, and expression levels of **(G)** PPAR-γ were examined by western blotting. **(H)** Cytotoxicity of naringin was studied by CCK8 assay in RAW 264.7 and IEC-6 cells. Data are expressed as the mean ± SD. Values in the normal control (NC) group are set to 100%, and other values are given relative to the NC group. *p <0.05 compared with NC group. *n* = 3 samples in western blotting experiments; *n* = 6 samples for other experiments. These blots are cropped, and the full-length blots are presented is in the Supplementary Figure S1.

The chemical structure of naringin is shown in Figure 1A. Naringin had no significant effect on body weight or food intake in normal mice (Figures 1B,C). Cytokine profiles [TNF-α, interleukins (IL)-1β, and interferon (INF)-γ] in colon tissue were not affected by naringin (Figures 1D–F). There was no significant effect on expression of PPAR-γ in colon tissue after gavage administration of 10 and 40 mg/kg naringin for 7 days, while the expression of PPAR-γ was decreased by 160 mg/kg naringin (Figure 1G). Naringin (5–160 μM, 12 h) did not significantly affect cell viability in RAW264.7 cells. Naringin (5–80 μM, 12 h) exerted no significant effects on cell viability in IEC6 cells, while 160 μM significantly inhibited cell viability (Figure 1H). According to our preliminary experiments and previous reports (Ahmad et al., 2015; Cao et al., 2018), the maximum doses of naringin were 40 mg/kg *in vivo* and 20 μM *in vitro*.

### Naringin Ameliorates DSS-Colitis Symptoms

**FIGURE 2:**
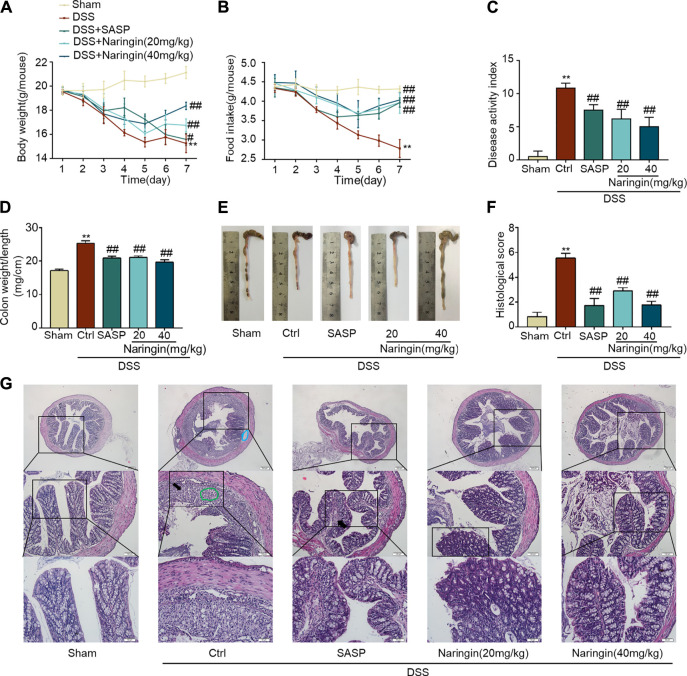
Naringin ameliorates DSS-colitis symptoms. Colitis symptoms were recorded after 7 days of naringin treatment. Effects of naringin on **(A)** body weight, **(B)** food intake, **(C)** DAI, **(D)** colon weight-to-length ratio, and **(E)** gross morphology of colon tissue. **(F)** Total histological score was calculated as the sum of epithelial damage and histological score. **(G)** H and E staining of mice colonic tissue. Scale bars, 200, 100, 50 μm. Green ring indicates damaged villi structure; black arrow indicates infiltration of inflammatory cells; blue ring indicates submucosal edema. Data are expressed as mean ± SD. Values in the sham group are set to 100%, and other values are given relative to the sham group. **p < 0.01 compared with sham group; ^##^ *p* < 0.01 compared with DSS-colitis group; *n* = 6 samples.

The significant weight loss, high colon weight-to-length ratio, decreased food intake and high DAI scores in the untreated model group showed that instillation of DSS to the colon resulted in reproducible colitis in mice (Figures 2A–D). The overall morphology of the colon is shown in Figure 2E. Histopathological damage was characteristically induced by DSS (Figures 2F,G). A variety of colitis symptoms, including damaged villi structure (green ring), infiltration of inflammatory cells (black arrow), submucosal edema (blue ring), and muscle fiber separation (blue arrow) were visible in the H and E staining (Figure 2G). Naringin and SASP reversed the pathological changes in colitis.

### Effects of Naringin on Inflammation and Apoptosis in DSS-Colitis

**FIGURE 3:**
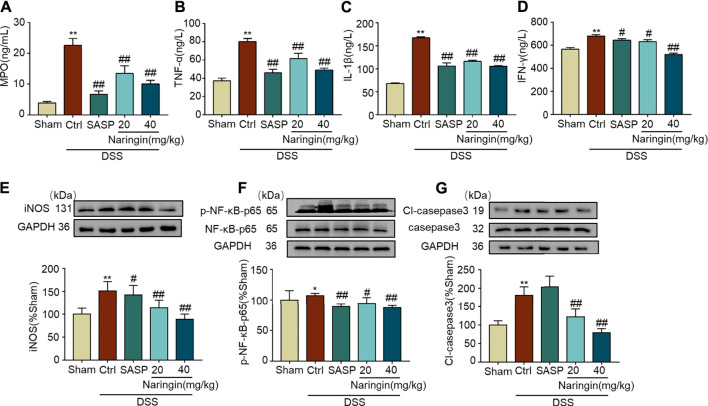
Effects of naringin on inflammation and apoptosis in DSS-colitis. Expression levels of the colonic cytokines **(A)** MPO, **(B)** TNF-α, **(C)** IL-1β, and **(D)** IFN-γ were determined by ELISA. Western blotting analysis of inflammation-related proteins **(E)** iNOS, **(F)** p-NF-κB-p65 (calculated as p-NF-κB-p65/NF-κB-p65); apoptosis-related protein **(G)** cl-caspase3, calculated as cl-caspase3/caspase3. Data are expressed as mean ± SD. Values in the sham group are set to 100% and other values are given relative to those in the sham group, *p < 0.05, **p < 0.01 compared with sham group; ^#^ *p* < 0.05, ^##^ *p* < 0.01 compared with DSS-colitis group; *n* = 3 samples in western blotting experiments; *n* = 6 samples for other experiments. These blots are cropped, and the full-length blots are presented is in the Supplementary Figures S2–S9.

As shown in Figures 3A–D, MPO activity and expression of pro-inflammatory factors (TNF-α, IL-1β, and INF-γ) were significantly increased in DSS-colitis, and naringin significantly reversed these changes. DSS-induced colitis up-regulated inflammation-related proteins (iNOS and p-NF-κB-p65) and an apoptosis-related protein cl-caspase3 expression. Finally, an attenuated trend was observed in groups treated with naringin (Figures 3E–G). These results confirmed that naringin has anti-inflammatory and anti-apoptotic activities in DSS-colitis.

### High-Throughput Transcriptome Sequencing and KEGG Pathway Enrichment Analysis

**FIGURE 4:**
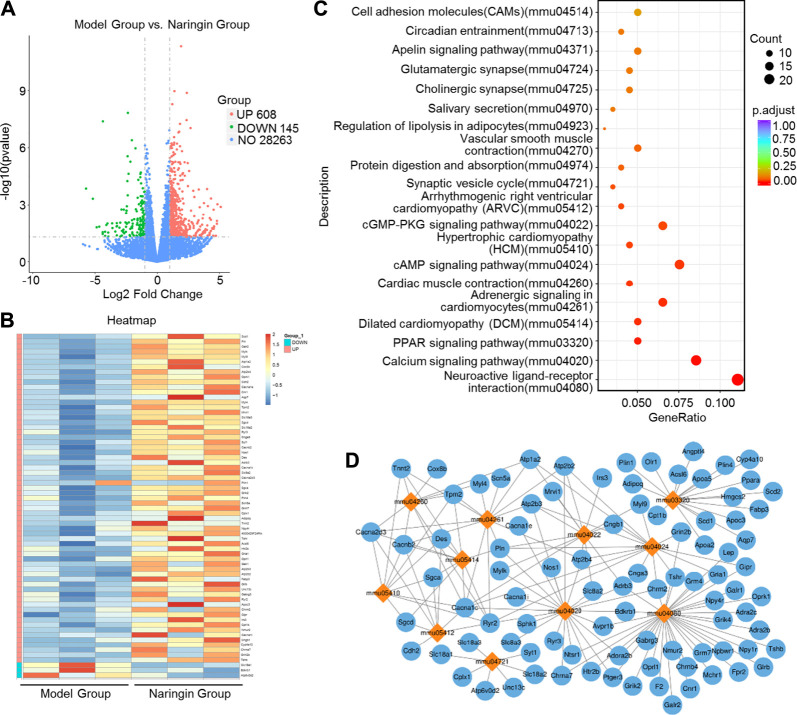
High-throughput transcriptome sequencing and KEGG pathway enrichment analysis. Naringin (40 mg/kg) was administered to mice by gavage once a day for seven consecutive days and total RNA was extracted from colonic epithelial cells. **(A)** Volcano plot of differentially expressed mRNAs in model group vs. naringin treatment group. **(B)** Heatmap of some representative genes. **(C)** The scatter plots showing the top 20 enriched KEGG pathways in model group vs. naringin group. The Rich factor is the ratio of differentially expressed gene numbers annotated in this pathway term to all gene number annotated in this pathway term. Greater rich factor means greater intensiveness. Q-value is corrected *p* value ranging from 0 to 1. **(D)** Target-pathway network including candidate 133 targets and 11 KEGG pathways.

**FIGURE 5:**
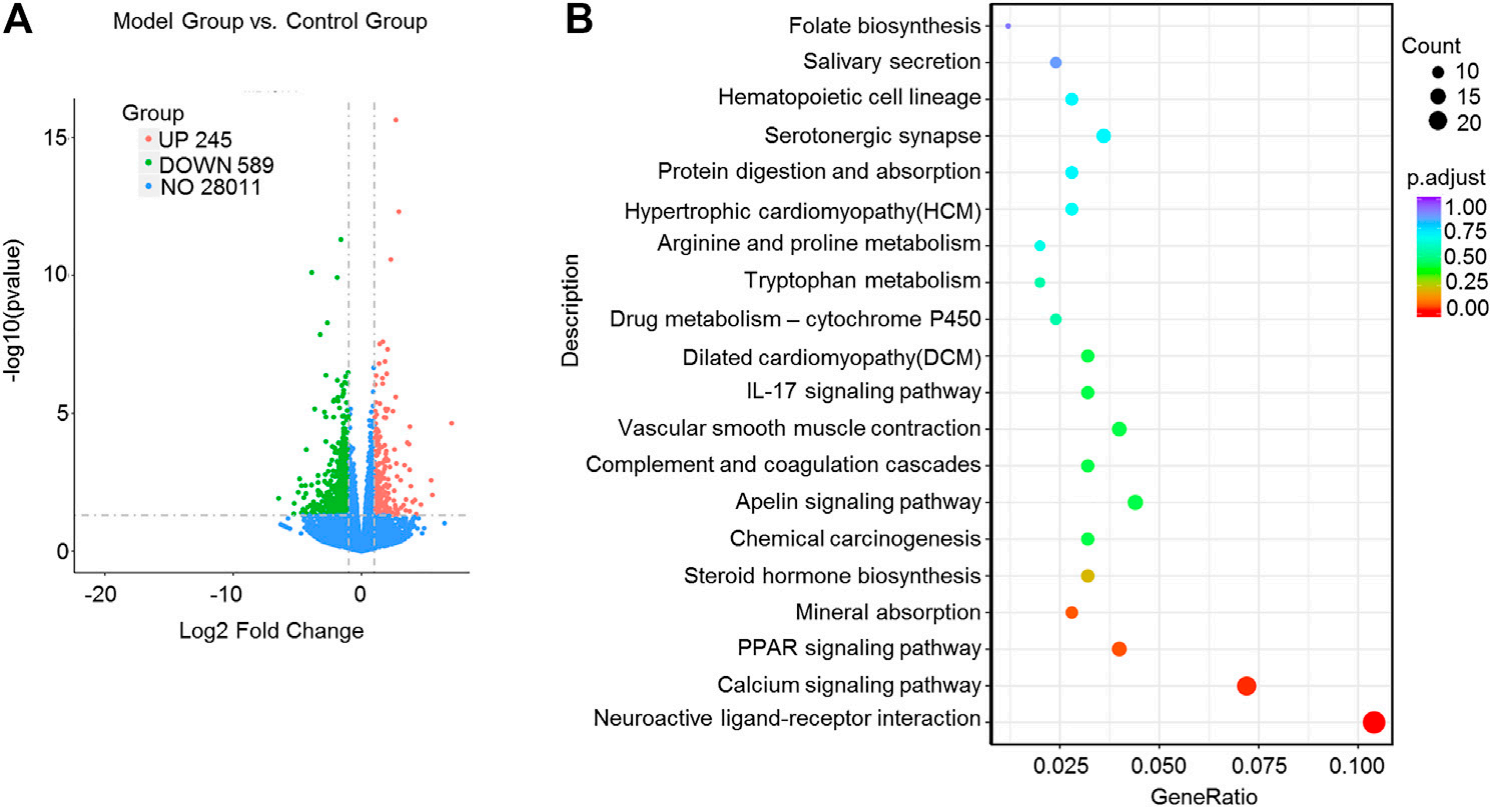
High-throughput transcriptome sequencing and KEGG pathway enrichment analysis. Naringin (40 mg/kg) was administered to mice by gavage once a day for seven consecutive days and total RNA was extracted from colonic epithelial cells. **(A)** Volcano plot of differentially expressed mRNAs in model group vs. control group. **(B)** The scatter plots showing the top 20 enriched KEGG pathways in model group vs. control group. The Rich factor is the ratio of differentially expressed gene numbers annotated in this pathway term to all gene number annotated in this pathway term. Greater rich factor means greater intensiveness. Q-value is corrected *p* value ranging from 0 to 1.

 We applied transcriptome sequencing to investigate the possible mechanisms of naringin in the treatment of colitis. A total of 753 differentially expressed mRNAs before and after naringin treatment were identified with fold changes > 2 and *p* < 0.05 and some representative genes were shown in heatmap (Figures 4A,B). In addition, compared with the control group, 834 differentially expressed mRNAs were observed in the model group (Figure 5A).

The KEGG enrichment results showed the top 20 enriched KEGG pathways in the model group vs. naringin treatment group (Figure 4C) and the control group vs. model group (Figure 5B). There are three common enriched KEGG pathways including the neuroactive ligand-receptor interaction, calcium signaling, and PPAR signaling among the top 20 enriched KEGG pathways between the model group vs. naringin treatment group and the control group vs. model group (Figure 4C, Figure 5B). Based on the genes contained in the top 11 pathways, we constructed a KEGG-target network of naringin-induced treatment including 133 targets (Figure 4D). Most targets were associated with different pathways, suggesting synergistic effects are involved in naringin-induced treatment. Therefore, naringin may target different pathways and targets.

### Effects of Naringin on PPAR-γ

**FIGURE 6:**
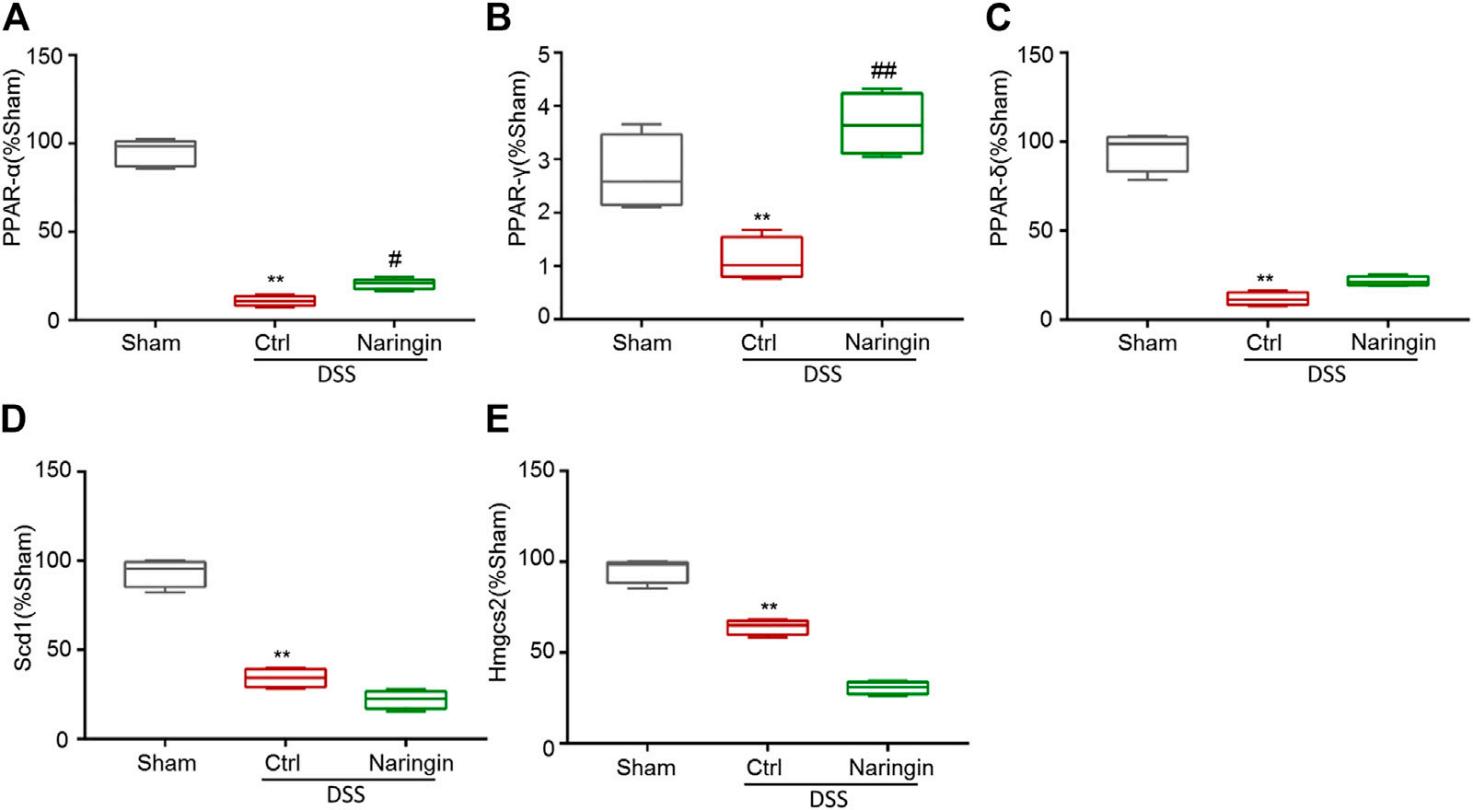
The effect of naringin on some mRNAs in PPAR signaling pathways detected by qRT-PCR. Naringin (40 mg/kg) was administered to mice by gavage once a day for seven consecutive days. The expression levels of some mRNAs in PPAR signaling pathways including **(A)** PPAR-α, **(B)** PPAR-γ, **(C)** PPAR-δ, **(D)** Scd1, and **(E)** Hmgcs2 were detected by qRT-PCR (means ± SD, *n* = 3). **p < 0.01 compared with sham group; ^#^ *p* < 0.05, ^##^ *p* < 0.01 compared with DSS-colitis group.

According to reference validation, we subsequently studied proteins in the PPAR signaling pathways to uncover naringin-induced treatment effects on colitis. We used qRT-PCR to detect the roles of some mRNAs in PPAR signaling pathways, including PPAR-α, PPAR-γ, PPAR-δ, Scd1, and Hmgcs2 in naringin-induced treatment effects (Figure 6). There were significant differences in the expressions of PPAR-α and PPAR-γ between the naringin treatment and DSS-colitis groups. The expressions of PPAR-δ, Scd1, and Hmgcs2 were not significantly affected by naringin in the colitis model.

**FIGURE 7:**
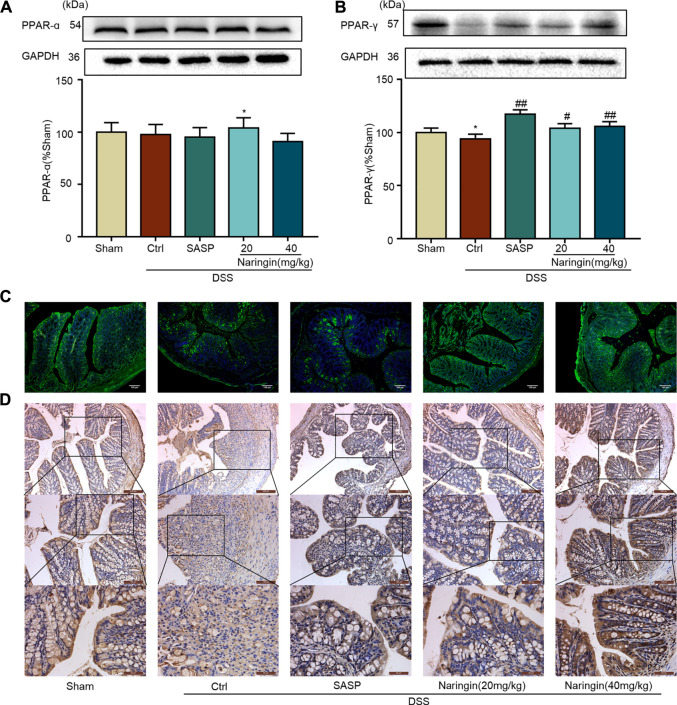
Effects of naringin on PPAR-γ expression in DSS-colitis. Naringin was administered to mice by gavage once a day for seven consecutive days. **(A)** Western blotting analysis of PPAR-α. **(B)** Western blotting analysis of PPAR-γ. **(C)** IF staining of PPAR-γ in colonic epithelium. Scale bars, 100 μm. **(D)** IHC staining of PPAR-γ in colonic epithelium. Scale bars, 200, 100, and 50 μm. Data are expressed as mean ± SD. Values in the sham group are set to 100% and other values are given relative to those in the sham group. **p < 0.01 compared with sham group; ^#^ *p* < 0.05, ^##^ *p* < 0.01 compared with DSS-colitis group; *n* = 3 samples in western blotting experiments; *n* = 6 samples for other experiments. These blots are cropped, and the full-length blots are presented is in the Supplementary Figures S10–S13.

We further studied the protein expressions of PPAR-α and PPAR-γ. Compared with the sham group, PPAR-γ expression was significantly decreased in DSS-colitis, but naringin treatment significantly stimulated PPAR-γ expression (Figures 7B–D). However, naringin treatment did not significantly affect PPAR-α protein expression (Figure 7A). The results suggest that PPAR-α and PPAR-γ may be involved in naringin-induced treatment effects, however, PPAR-γ plays a more important role.

**FIGURE 8:**
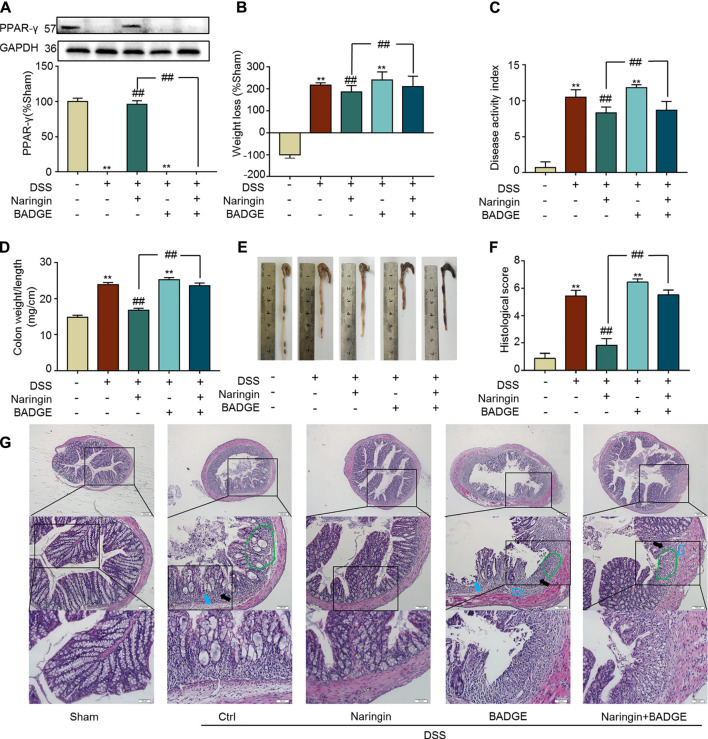
Effects of PPAR-γ inhibitor BAGDE (30 mg/kg) on naringin induced treatment. **(A)** PPAR-γ expression in the presence of PPAR-γ inhibitor BAGDE. Effects of naringin (40 mg/kg) on **(B)** weight loss, **(C)** DAI, **(D)** colon weight-to-length ratio and **(E)** gross morphology of colon tissue. **(F)** Total histological score was calculated as the sum of epithelial damage and histological score. **(G)** H and E staining analysis of the aggravated symptoms with BADGE. Scale bars, 200, 100, and 50 μm. Green ring indicates damaged villi structure; black arrow indicates infiltration of inflammatory cells; blue ring indicates submucosal edema, blue arrow indicates muscle fiber separation. Data are expressed as mean ± SD. Values in the sham group are set to 100% and other values are given relative to those in the sham group. **p < 0.01 compared with sham group; ^##^ *p* < 0.01 compared with DSS-colitis group; *n* = 3 samples in western blotting experiments; *n* = 6 samples for other experiments. These blots are cropped, and the full-length blots are presented is in the Supplementary Figures S14–S15.

Next, we examined the role of PPAR-γ for naringin-induced treatment effects. *In vivo*, the PPAR-γ inhibitor BADGE was used to investigate the role of PPAR-γ in naringin-induced improvement of colitis. As shown in Figure 8A, PPAR-γ expression was significantly inhibited by BADGE, and BADGE significantly exacerbated colitis symptoms including weight loss (Figure 8B), high DAI, increased colon weight-to-length ratio, and histological score (Figures 8C,D,F). The overall morphology of the colon is shown in Figure 8E. Every colitis symptom was reversed by naringin, but this affect was significantly inhibited by BADGE.

**FIGURE 9:**
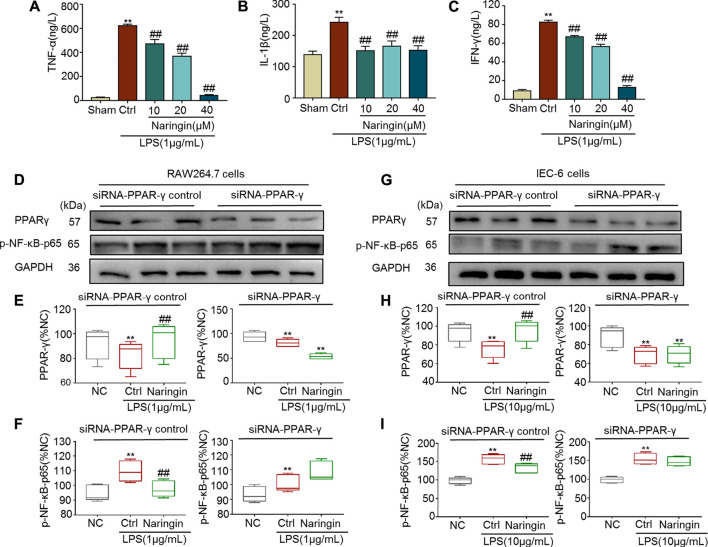
The anti-inflammatory effect of naringin (20 μM) was eliminated by siRNA targeting PPAR-γ. Expression levels of the colonic cytokines **(A)** TNF-α, **(B)** IL-1β, **(C)** IFN-γ in DSS-induced RAW264.7 cells were determined by ELISA. Expression of PPAR-γ and p-NF-κB-p65 in the absence and presence of siRNA targeting PPAR-γ in **(D–F)** RAW 264.7 and **(G–I)** IEC-6 cells. Data are expressed as mean ± SD. Values in corresponding sham or normal control (NC) group are set to 100% and other values are given relative to the control values. **p < 0.01 compared with sham/NC group; ^##^ *p* < 0.01 compared with DSS/LPS control group or as indicated; *n* = 3 samples in western blotting experiments; *n* = 6 samples for other experiments. These blots are cropped, and the full-length blots are presented is in the Supplementary Figures S16–S21.

Both RAW264.7 and IEC-6 cells were used to confirm the stimulatory effects of naringin on PPAR-γ. In RAW264.7 cells, pro-inflammatory cytokines, including TNF-α, IL-1β, and INF-γ, were significantly increased by LPS, and the increase was reversed by naringin (Figures 9A–C). P-NF-κB-p65, a target gene of PPAR-γ, was also significantly increased by LPS (Figures 9D–F). The LPS induced increase in p-NF-κB-p65 was significantly reversed by naringin, but was significantly inhibited by siRNA targeting PPAR-γ. Similar results were also obtained using IEC-6 cells (Figures 9G–I). These results suggested that PPAR-γ is a potential target of naringin.

### Effects of Naringin on TNBS-Colitis

**FIGURE 10:**
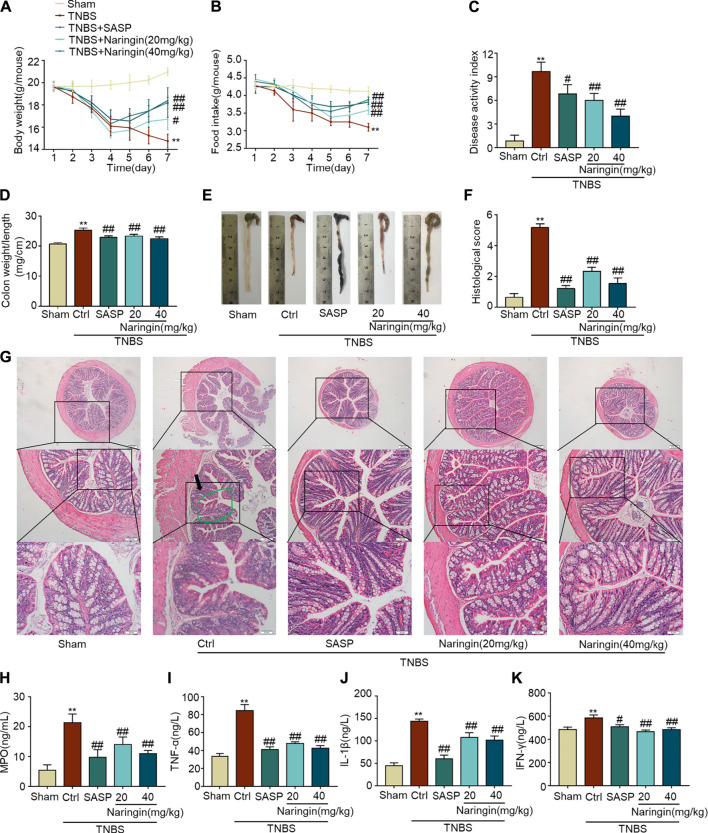
Effects of naringin on TNBS-colitis. Naringin was administered to mice by gavage once a day for seven consecutive days. Effects of naringin on **(A)** body weight, **(B)** food intake, **(C)** DAI, **(D)** colon weight-to-length ratio, and **(E)** gross morphology of colon tissue. **(F)** Total histological score was calculated as the sum of epithelial damage and histological score. **(G)** H and E staining of mice colonic tissue. Scale bars, 200, 100, and 50 μm. Green ring indicates damaged villi structure; black arrow indicates infiltration of inflammatory cells. Expression levels of the colonic cytokines **(H)** MPO, **(I)** TNF-α, **(J)** IL-1β, and **(K)** IFN-γ were determined by ELISA. Data are expressed as mean ± SD. Values in the sham group are set to 100% and other values are given relative to those in the sham group. **p < 0.01 compared with sham group; ^#^ *p* < 0.05, ^##^ *p* < 0.01 compared with TNBS-colitis group; *n* = 6 samples.

A TNBS-colitis model was also established to study the effects of naringin. Compared with the sham group, significant weight loss, high colon weight-to-length ratio, lower food intake, high DAI scores, and increased histological score were observed in the untreated colitis model group (Figures 10A–D,F). The overall morphology of the colon is shown in Figure 10E. Naringin significantly reversed these colitis symptoms. Higher levels of MPO activity and the expression of pro-inflammatory cytokines (IL-1β, INF-γ, and TNF-α) were also reversed by naringin treatment (Figures 10H–K). Naringin ameliorated the above colitis symptoms as evidenced by H and E staining (Figure 10G). Damaged villi structure (green ring), infiltration of inflammatory cells (black arrow), and submucosal edema (blue ring) shown in H and E staining were alleviated by naringin.

**FIGURE 11:**
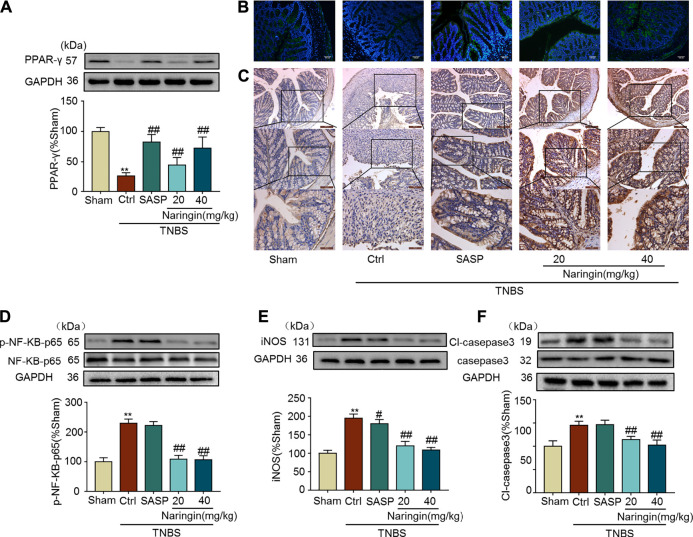
Effects of naringin on PPAR-γ expression, inflammation and apoptosis in TNBS-colitis mouse model. **(A)** Western blotting analysis of PPAR-γ. **(B)** IF staining of PPAR-γ in colonic epithelium. Scale bars, 100 μm. **(C)** IHC staining of PPAR-γ in colonic epithelium. Scale bars, 200, 100, and 50 μm. Western blotting analysis of inflammation-related proteins **(D)** p-NF-κB-p65 (calculated as p-NF-κB-p65/ NF-κB-p65), **(E)** iNOS; apoptosis-related protein **(F)** cl-caspase3, calculated as cl-caspase3/caspase3. Data are expressed as mean ± SD. Values in the sham group are set to 100% and other values are given relative to those in the sham group. **p < 0.01 compared with sham group; ^#^ *p* < 0.05, ^##^ *p* < 0.01 compared with TNBS-colitis group; *n* = 3 samples in western blotting experiments; *n* = 6 samples for other experiments. These blots are cropped, and the full-length blots are presented is in the Supplementary Figures S22–S31.

Naringin can reverse the increasing expression of inflammation-related proteins (iNOS and p-NF-κB-p65), and apoptosis-related protein cl-caspase3 in TNBS-colitis (Figures 11D–F). Furthermore, decreased PPAR-γ expression in TNBS-colitis was also stimulated by gastric administration of naringin (Figures 11A–C). In accordance with our DSS-colitis results, naringin also significantly alleviated TNBS-colitis.

## Discussion

In this study, we examined the effects and detailed mechanisms of naringin on mouse colitis. We showed that naringin had therapeutic effects on both DSS- and TNBS-induced colitis. Colitis symptoms, including neutrophil infiltration, cytokine profiles, and epithelial barrier dysfunction were significantly ameliorated by naringin. In this study, PPAR-γ was found to be an important target for naringin-induced improvement of colitis.

Based on RNA-seq analysis, 753 mRNAs were identified that differed between untreated colitis and naringin treated groups. We identified 11 important pathways involved using a KEGG-target network. Some of these pathways have previously been shown to be involved in colitis. For example, the disruption of Ca^2+^ homeostasis is associated with colonic dysmotility in UC (Wang et al., 2016a). cAMP/PKA-dependent and independent pathways can reduce the expression of inflammatory mediators and attenuate p38 phosphorylation to treat TNBS-colitis (Sun et al., 2017). The activation of PPAR-α, PPAR-δ, and Scd1 has been shown to play a role in DSS-colitis, and Hmgcs2 has an effect in TNBS-colitis (Bassaganya-Riera et al., 2004; Wang et al., 2016b; Wang et al., 2017; Liu et al., 2018). Cnr1, part of the neuroactive ligand-receptor interaction, is reported to be associated with IBD symptoms (Storr et al., 2010). The expression of Mylk in the calcium signaling pathway is a molecular marker of intestinal fibrosis, a critical complication of CD (Rodansky et al., 2015). In our study, PPAR-α, PPAR-γ, PPAR-δ, Scd1, and Hmgcs2 involved in PPAR signaling pathways were selected for preliminary research. Although we found that PPAR-γ is the main target for naringin-induced improvement of colitis, there are multiple targets involved. The other potential targets of naringin will be studied in the future.

PPAR-γ is a transcription factor that plays an important role in anti-inflammation, antioxidant, and phagocyte-mediated cleanup processes and is highly expressed in colonic epithelium and adipose tissue (Lefebvre et al., 1999; Dubuquoy et al., 2002). Several studies have demonstrated that expression of PPAR-γ is reduced in UC patients (Dubuquoy et al., 2003; Pedersen and Brynskov, 2010). In the present study, PPAR-γ expression was significantly decreased in both DSS- and TNBS-induced colitis. Reduced PPAR-γ expression may lead to an increase in expression of nuclear transcription factor NF-κB. PPAR-γ can directly bind to NF-κB p50/NF-κB p65 dimer and inhibit the degradation of IκBα, thereby blocking the activation of NF-κB and its nuclear translocation (Chen et al., 2014). Gavage administration of naringin significantly alleviated colitis symptoms in two animal models. Increased MPO activity and higher levels of pro-inflammatory factors (TNF-α, IL-1β, and INF-γ) in colitis were significantly reversed by naringin. High expression of the inflammation-related protein iNOS in colitis was significantly decreased by naringin. Naringin also significantly reduced cl-caspase3 expression to maintain epithelial barrier. Naringin induced alleviation of colitis symptoms was abolished by PPAR-γ inhibitor BADGE, which suggests that PPAR-γ may be a target for naringin induced therapeutic effects on colitis.

In RAW264.7 cells, higher levels of pro-inflammatory factors (TNF-α, IL-1β, and INF-γ) were significantly reversed by naringin. In IEC-6 cells and RAW264.7 cells, LPS-induced high expression of NF-κB-p65 was significantly reduced by naringin, and the reduction was inhibited by siRNA targeting PPAR-γ. All these results suggests that naringin exert treatment effects on colitis through inducing PPAR-γ activation. It has been reported that some PPAR-γ agonists, such as rosiglitazone, pioglitazone, exert inhibitory effects on inflammatory responses (Nitta et al., 2013; Zhou et al., 2021). In this study, we aimed to search and identify natural products which may serve as PPAR-γ agonists. Compared with chemical drugs, naringin is a monomer isolated from citrus fruit species with better safety.

The two animal models of colitis were used in this study to confirm the treatment effects of naringin. DSS-colitis is a type of acute injury and it is caused by disrupting the integrity of the mucosal barrier. TNBS-colitis is characterized by T-cell-mediated immunity against haptenized proteins and luminal antigens (Gadaleta et al., 2017). More or less, DSS-induced intestinal inflammation is mainly mediated by an excessive Th1 T-cell response (CD-like IBD), while TNBS-induced intestinal inflammation is a Th2-mediated inflammation (UC-like IBD) (Strober et al., 2002). PPAR-γ activation can inhibit the inflammatory response by inhibiting NF-κB. It is also known that PPAR-γ upregulating tight junction improves the integrity of intestinal mucosal barrier (Huang et al., 2020). In this study, naringin had a good therapeutic effect on both types of colitis through activating PPAR-γ. We used transcriptome sequencing to detect the expression of genes only in naringin treated DSS-induced C57BL/6 mice. Whether the mechanisms of naringin treatment of TNBS-colitis is the same as that of DSS-colitis still needs further investigation.

In conclusion, we uncovered the drug target net of naringin for alleviating DSS- and TNBS-induced colitis. We established a KEGG-target network to explore detailed mechanisms involved in naringin-induced amelioration of colitis symptoms. PPAR-γ may be a main potential target of naringin. There are still some limitations for our study, for example, the interaction between naringin and PPAR-γ is not clearly studied. The future plan is to extensively explore the interaction between naringin and PPAR-γ, including the exact binding site of PPAR-γ for naringin. The chemical structure of monomers isolated from Chinese medicinal plants is complex, and they can exert pharmacological effects through multiple targets. RNA-seq may be a good method to help researchers to construct the drug target net for monomers isolated from Chinese medicinal plants.

## Data Availability

The datasets presented in this study can be found in online repositories. The names of the repository/repositories and accession number(s) can be found below: BioProject accession: PRJNA741857.

## Abbreviations

BSA, bovine serum albumin; CD, Crohn’s disease; Cl-caspase3, cleaved caspase3; DAI, disease activity index; DSS, dextran sodium sulphate; H and E, hematoxylin-eosin; IBD, inflammatory bowel disease; IF, immunofluorescent; IHC, immunohistochemical; IL, interleukins; INF, interferon; LPS, lipopolysaccharide; PBS, phosphate-buffered saline; PBST, PBS containing Tween-20; p-NF-κB-p65, phosphorylated NF-κB-p65; PPAR, peroxisome proliferator-activated receptor; qRT-PCR, quantitative real-time polymerase chain reaction; RNA-seq, RNA sequencing; SASP, Sulfasalazine; siRNA, interfering RNA; TNBS, 2, 4, 6-trinitrobenzene sulfonic acid; TNF, tumor necrosis factor; UC, ulcerative colitis.

## References

[B1] Abraham B. P. Ahmed T. Ali T. (2017). Inflammatory Bowel Disease: Pathophysiology and Current Therapeutic Approaches. Handb Exp. Pharmacol. 239, 115–146. 10.1007/164_2016_122 28233184

[B2] Ahmad S. F. Attia S. M. Bakheet S. A. Zoheir K. M. Ansari M. A. Korashy H. M. (2015). Naringin Attenuates the Development of Carrageenan-Induced Acute Lung Inflammation through Inhibition of NF-Κb, STAT3 and Pro-inflammatory Mediators and Enhancement of IκBα and Anti-inflammatory Cytokines. Inflammation 38, 846–857. 10.1007/s10753-014-9994-y 25117567

[B3] Ali M. M. El Kader M. A. (2004). The Influence of Naringin on the Oxidative State of Rats with Streptozotocin-Induced Acute Hyperglycaemia. Z. Naturforsch C J. Biosci. 59, 726–733. 10.1515/znc-2004-9-1018 15540607

[B4] Bassaganya-Riera J. Reynolds K. Martino-Catt S. Cui Y. Hennighausen L. Gonzalez F. (2004). Activation of PPAR Gamma and delta by Conjugated Linoleic Acid Mediates protection from Experimental Inflammatory Bowel Disease. Gastroenterology 127, 777–791. 10.1053/j.gastro.2004.06.049 15362034

[B5] Bolger A. M. Lohse M. Usadel B. (2014). Trimmomatic: a Flexible Trimmer for Illumina Sequence Data. Bioinformatics 30, 2114–2120. 10.1093/bioinformatics/btu170 24695404 PMC4103590

[B6] Cao H. Liu J. Shen P. Cai J. Han Y. Zhu K. (2018). Protective Effect of Naringin on DSS-Induced Ulcerative Colitis in Mice. J. Agric. Food Chem. 66, 13133–13140. 10.1021/acs.jafc.8b03942 30472831

[B7] Chen K. Li J. Wang J. Xia Y. Dai W. Wang F. (2014). Erratum to "15-Deoxy-Γ12,14-Prostaglandin J2 Reduces Liver Impairment in a Model of ConA-Induced Acute Hepatic Inflammation by Activation of PPARγ and Reduction in NF-Κb Activity". PPAR Res. 2014, 864839. 10.1155/2014/864839 25435143 PMC4238265

[B8] Conesa A. Madrigal P. Tarazona S. Gomez-Cabrero D. Cervera A. McPherson A. (2016). A Survey of Best Practices for RNA-Seq Data Analysis. Genome Biol. 17, 13. 10.1186/s13059-016-0881-8 26813401 PMC4728800

[B9] Dong J.-y. Xia K.-j. Liang W. Liu L.-l. Yang F. Fang X.-s. (2020). Ginsenoside Rb1 Alleviates Colitis in Mice via Activation of Endoplasmic Reticulum-Resident E3 Ubiquitin Ligase Hrd1 Signaling Pathway. Acta Pharmacol. Sin. 10.1038/s41401-020-00561-9 PMC8379258 33268823

[B10] Dubuquoy L. Dharancy S. Nutten S. Pettersson S. Auwerx J. Desreumaux P. (2002). Role of Peroxisome Proliferator-Activated Receptor Gamma and Retinoid X Receptor Heterodimer in Hepatogastroenterological Diseases. Lancet 360, 1410–1418. 10.1016/s0140-6736(02)11395-x 12424006

[B11] Dubuquoy L. Jansson E. A. Deeb S. Rakotobe S. Karoui M. Colombel J. F. (2003). Impaired Expression of Peroxisome Proliferator-Activated Receptor Gamma in Ulcerative Colitis. Gastroenterology 124, 1265–1276. 10.1016/s0016-5085(03)00271-3 12730867

[B12] Feng P. P. Fang X. S. Zhao S. H. Fu J. Y. Zhang H. T. Yi Y. L. (2020). Salvianolic Acid B Decreases Interleukin-1β-Induced Colitis Recurrence in Mice. Chin. Med. J. (Engl) 133, 1436–1444. 10.1097/cm9.0000000000000773 32472783 PMC7339324

[B13] Gadaleta R. M. Garcia-Irigoyen O. Moschetta A. (2017). Exploration of Inflammatory Bowel Disease in Mice: Chemically Induced Murine Models of Inflammatory Bowel Disease (IBD). Curr. Protoc. Mouse Biol. 7, 13–28. 10.1002/cpmo.20 28252200

[B14] Gao F. Liu X. Wu X. P. Wang X. L. Gong D. Lu H. (2012). Differential DNA Methylation in Discrete Developmental Stages of the Parasitic Nematode *Trichinella spiralis* . Genome Biol. 13, R100. 10.1186/gb-2012-13-10-r100 23075480 PMC4053732

[B15] Grivennikov S. I. (2013). Inflammation and Colorectal Cancer: Colitis-Associated Neoplasia. Semin. Immunopathol 35, 229–244. 10.1007/s00281-012-0352-6 23161445 PMC3568220

[B16] Holmer A. Singh S. (2019). Overall and Comparative Safety of Biologic and Immunosuppressive Therapy in Inflammatory Bowel Diseases. Expert Rev. Clin. Immunol. 15, 969–979. 10.1080/1744666X.2019.1646127 31322018 PMC6813772

[B17] Huang Y. Wang C. Tian X. Mao Y. Hou B. Sun Y. (2020). Pioglitazone Attenuates Experimental Colitis-Associated Hyperalgesia through Improving the Intestinal Barrier Dysfunction. Inflammation 43, 568–578. 10.1007/s10753-019-01138-3 31989391 PMC7170986

[B18] Jeon S. M. Bok S. H. Jang M. K. Lee M. K. Nam K. T. Park Y. B. (2001). Antioxidative Activity of Naringin and Lovastatin in High Cholesterol-Fed Rabbits. Life Sci. 69, 2855–2866. 10.1016/s0024-3205(01)01363-7 11720089

[B19] Kaplan G. G. Windsor J. W. (2021). The Four Epidemiological Stages in the Global Evolution of Inflammatory Bowel Disease. Nat. Rev. Gastroenterol. Hepatol. 18, 56–66. 10.1038/s41575-020-00360-x 33033392 PMC7542092

[B20] Kihara N. de la Fuente S. G. Fujino K. Takahashi T. Pappas T. N. Mantyh C. R. (2003). Vanilloid Receptor-1 Containing Primary Sensory Neurones Mediate Dextran Sulphate Sodium Induced Colitis in Rats. Gut 52, 713–719. 10.1136/gut.52.5.713 12692058 PMC1773638

[B21] Kilkenny C. Browne W. Cuthill I. C. Emerson M. Altman D. G. (2010). Animal Research: Reporting *In Vivo* Experiments: the ARRIVE Guidelines. J. Physiol. 588, 2519–2521. 10.1113/jphysiol.2010.192278 20634180 PMC2916981

[B22] Kukurba K. R. Montgomery S. B. (2015). RNA Sequencing and Analysis. Cold Spring Harb Protoc. 2015, 951–969. 10.1101/pdb.top084970 25870306 PMC4863231

[B23] Lefebvre M. Paulweber B. Fajas L. Woods J. McCrary C. Colombel J. F. (1999). Peroxisome Proliferator-Activated Receptor Gamma Is Induced during Differentiation of colon Epithelium Cells. J. Endocrinol. 162, 331–340. 10.1677/joe.0.1620331 10467224

[B24] Liu X. Yu X. Xu X. Zhang X. Zhang X. (2018). The Protective Effects of Poria Cocos-Derived Polysaccharide CMP33 against IBD in Mice and its Molecular Mechanism. Food Funct. 9, 5936–5949. 10.1039/c8fo01604f 30378628

[B25] Melgar S. Karlsson A. Michaëlsson E. (2005). Acute Colitis Induced by Dextran Sulfate Sodium Progresses to Chronicity in C57BL/6 but Not in BALB/c Mice: Correlation between Symptoms and Inflammation. Am. J. Physiol. Gastrointest. Liver Physiol. 288, G1328–G1338. 10.1152/ajpgi.00467.2004 15637179

[B26] Nitta Y. Tahara N. Tahara A. Honda A. Kodama N. Mizoguchi M. (2013). Pioglitazone Decreases Coronary Artery Inflammation in Impaired Glucose Tolerance and Diabetes Mellitus: Evaluation by FDG-PET/CT Imaging. JACC Cardiovasc. Imaging 6, 1172–1182. 10.1016/j.jcmg.2013.09.004 24229770

[B27] Pedersen G. Brynskov J. (2010). Topical Rosiglitazone Treatment Improves Ulcerative Colitis by Restoring Peroxisome Proliferator-Activated Receptor-Gamma Activity. Am. J. Gastroenterol. 105, 1595–1603. 10.1038/ajg.2009.749 20087330

[B28] Rodansky E. S. Johnson L. A. Huang S. Spence J. R. Higgins P. D. (2015). Intestinal Organoids: a Model of Intestinal Fibrosis for Evaluating Anti-fibrotic Drugs. Exp. Mol. Pathol. 98, 346–351. 10.1016/j.yexmp.2015.03.033 25828392 PMC5915372

[B29] Sairenji T. Collins K. L. Evans D. V. (2017). An Update on Inflammatory Bowel Disease. Prim. Care 44, 673–692. 10.1016/j.pop.2017.07.010 29132528

[B30] Storr M. Emmerdinger D. Diegelmann J. Pfennig S. Ochsenkühn T. Göke B. (2010). The Cannabinoid 1 Receptor (CNR1) 1359 G/A Polymorphism Modulates Susceptibility to Ulcerative Colitis and the Phenotype in Crohn's Disease. PloS one 5, e9453. 10.1371/journal.pone.0009453 20195480 PMC2829088

[B31] Strober W. Fuss I. J. Blumberg R. S. (2002). The Immunology of Mucosal Models of Inflammation. Annu. Rev. Immunol. 20, 495–549. 10.1146/annurev.immunol.20.100301.064816 11861611

[B32] Sui J. Zhang C. Fang X. Wang J. Li Y. Wang J. (2020). Dual Role of Ca2+-Activated Cl- Channel Transmembrane Member 16A in Lipopolysaccharide-Induced Intestinal Epithelial Barrier Dysfunction *In Vitro* . Cell Death Dis 11, 404. 10.1038/s41419-020-2614-x 32472021 PMC7260209

[B33] Sun W. Cai Y. Zhang X. X. Chen H. Lin Y. D. Li H. (2017). Osthole Pretreatment Alleviates TNBS-Induced Colitis in Mice via Both cAMP/PKA-dependent and Independent Pathways. Acta Pharmacol. Sin 38, 1120–1128. 10.1038/aps.2017.71 28603288 PMC5547558

[B34] Wang L. Xie H. Xu L. Liao Q. Wan S. Yu Z. (2017). rSj16 Protects against DSS-Induced Colitis by Inhibiting the PPAR-α Signaling Pathway. Theranostics 7, 3446–3460. 10.7150/thno.20359 28912887 PMC5596435

[B35] Wang R. Gu X. Dai W. Ye J. Lu F. Chai Y. (2016). A Lipidomics Investigation into the Intervention of Celastrol in Experimental Colitis. Mol. Biosyst. 12, 1436–1444. 10.1039/c5mb00864f 27021137 PMC6338340

[B36] Wang Y. Li J. X. Ji G. J. Zhai K. Wang H. H. Liu X. G. (2016). The Involvement of Ca(2+) Signal Pathways in Distal Colonic Myocytes in a Rat Model of Dextran Sulfate Sodium-Induced Colitis. Chin. Med. J. (Engl) 129, 1185–1192. 10.4103/0366-6999.181968 27174327 PMC4878164

[B37] Xiong Y. Shi L. Wang L. Zhou Z. Wang C. Lin Y. (2017). Activation of Sirtuin 1 by Catalpol-Induced Down-Regulation of microRNA-132 Attenuates Endoplasmic Reticulum Stress in Colitis. Pharmacol. Res. 123, 73–82. 10.1016/j.phrs.2017.05.030 28655643

[B38] Yanai H. Hanauer S. B. (2011). Assessing Response and Loss of Response to Biological Therapies in IBD. Am. J. Gastroenterol. 106, 685–698. 10.1038/ajg.2011.103 21427713

[B39] Zhang Y. S. Li Y. Wang Y. Sun S. Y. Jiang T. Li C. (2016). Naringin, a Natural Dietary Compound, Prevents Intestinal Tumorigenesis in Apc (Min/+) Mouse Model. J. Cancer Res. Clin. Oncol. 142, 913–925. 10.1007/s00432-015-2097-9 26702935 PMC11819250

[B40] Zhou J. P. Yang X. N. Song Y. Zhou F. Liu J. J. Hu Y. Q. (2021). Rosiglitazone Alleviates Lipopolysaccharide-Induced Inflammation in RAW264.7 Cells via Inhibition of NF-Κb and in a PPARγ-dependent Manner. Exp. Ther. Med. 22, 743. 10.3892/etm.2021.10175 34055059 PMC8138265

