# Supplementary material for: Naringin Exerts Therapeutic Effects on Mice Colitis: A Study Based on Transcriptomics Combined With Functional Experiments
Source: Front Pharmacol. 2021 Aug 24;12:729414. doi: 10.3389/fphar.2021.729414 (PMC8421552; doi:10.3389/fphar.2021.729414)
Supplement: Supplementary file 1 [file DataSheet1.PDF]

Supplementary Material

Supplementary Figures

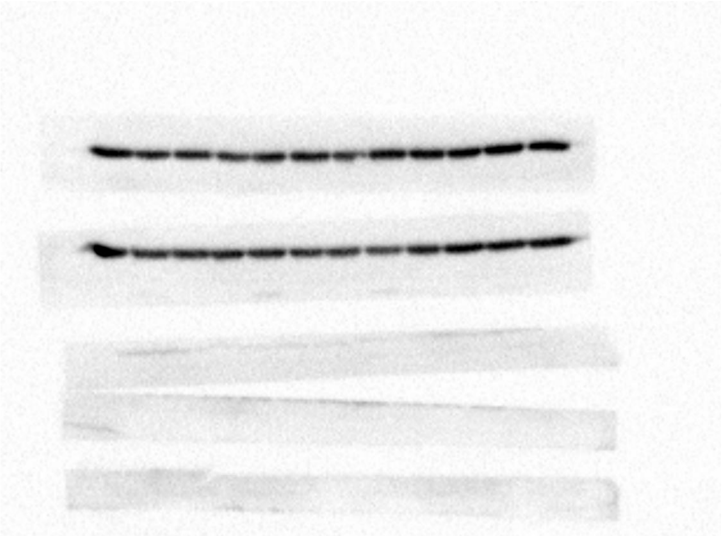

Supplementary Figure 1

The first blot:**GAPDH**: 36kDa

The second blot:**PPARG**: 57kDa

C group: representative image

The sample oder: A group :Naringin(0mg/kg), Naringin(10mg/kg), Naringin(40mg/kg), Naringin(160mg/kg),B group:Naringin(0mg/kg), Naringin(10mg/kg), Naringin(40mg/kg), Naringin(160mg/kg),C group:Naringin(0mg/kg), Naringin(10mg/kg), Naringin(40mg/kg), Naringin(160mg/kg)

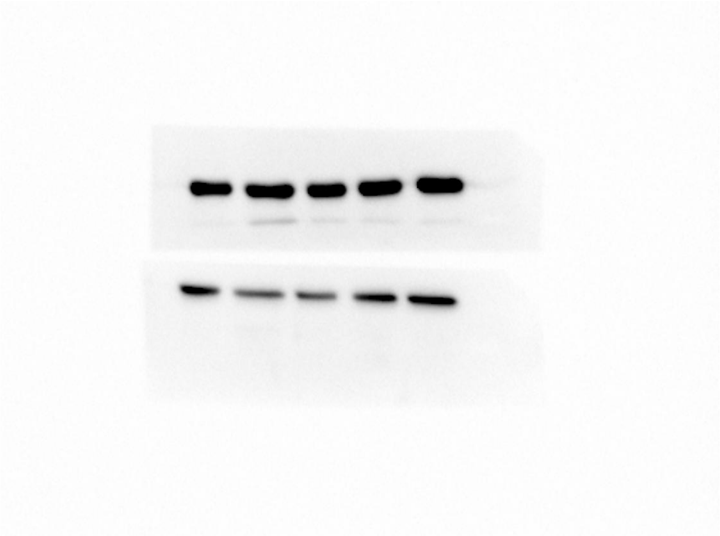

Supplementary Figure 2

GAPDH: 36kDa

The above blot:representative image

The sample oder: Sham, Control, SASP, Naringin(20mg/kg), Naringin(40mg/kg)

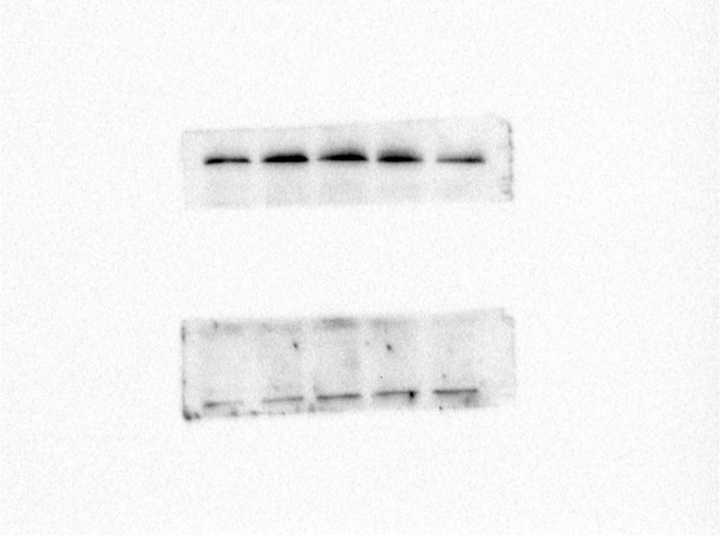

Supplementary Figure 3

iNOS: 131kDa

The above blot:representative image

The sample oder: Sham, Control, SASP, Naringin(20mg/kg), Naringin(40mg/kg)

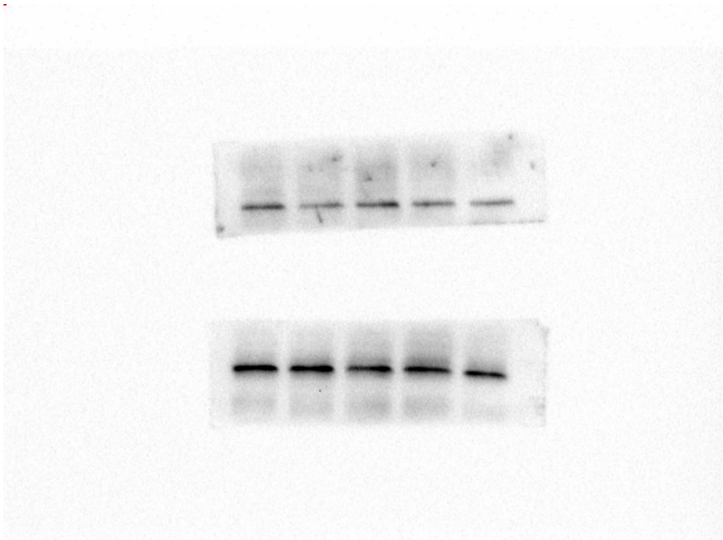

Supplementary Figure 4

NF-kB-p65: 65kDa

The following blot: representative image

The sample oder: Sham, Control, SASP, Naringin(20mg/kg), Naringin(40mg/kg)

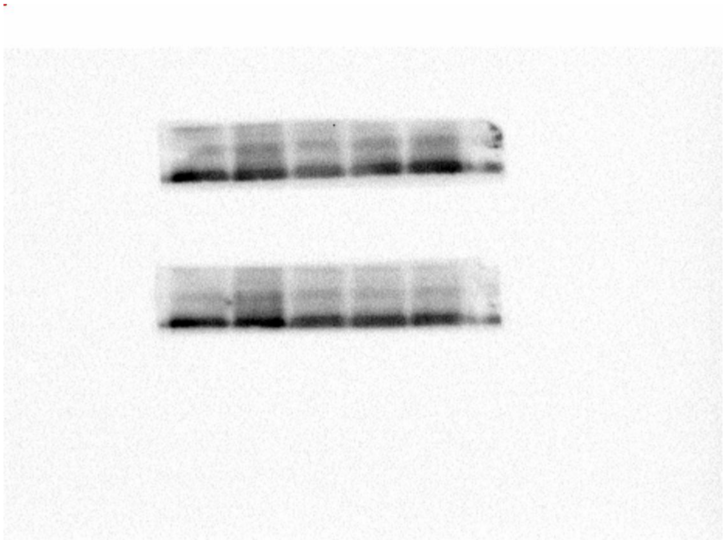

Supplementary Figure 5

p-NF-kB-p65: 65kDa

The following blot: representative image

The sample oder: Sham, Control, SASP, Naringin(20mg/kg), Naringin(40mg/kg)

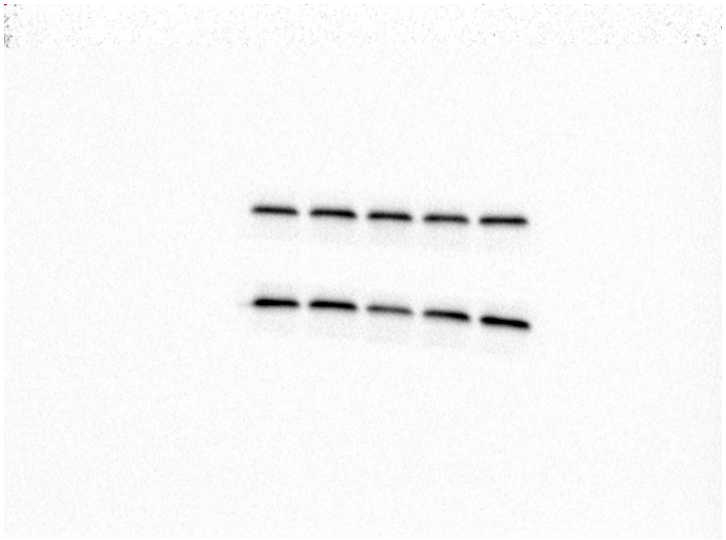

Supplementary Figure 6

GAPDH: 36kDa

The above blot: representative image

The sample oder: Sham, Control, SASP, Naringin(20mg/kg), Naringin(40mg/kg)

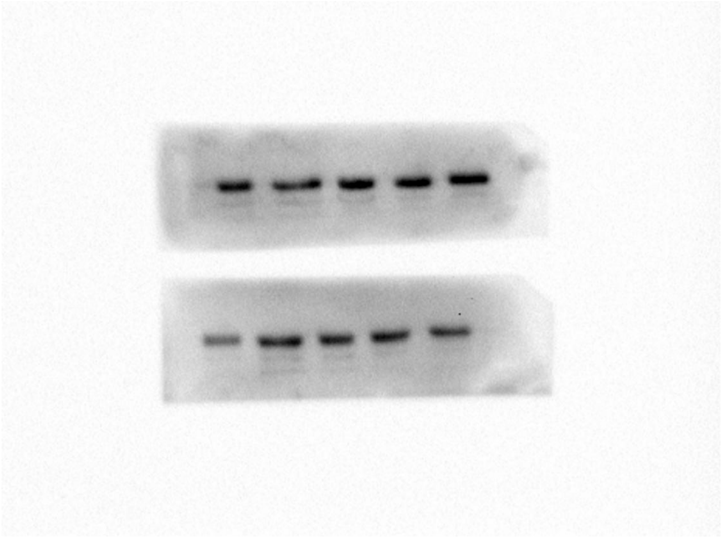

Supplementary Figure 7

caspase3: 32kDa

The following blot: representative image

The sample oder: Sham, Control, SASP, Naringin(20mg/kg), Naringin(40mg/kg)

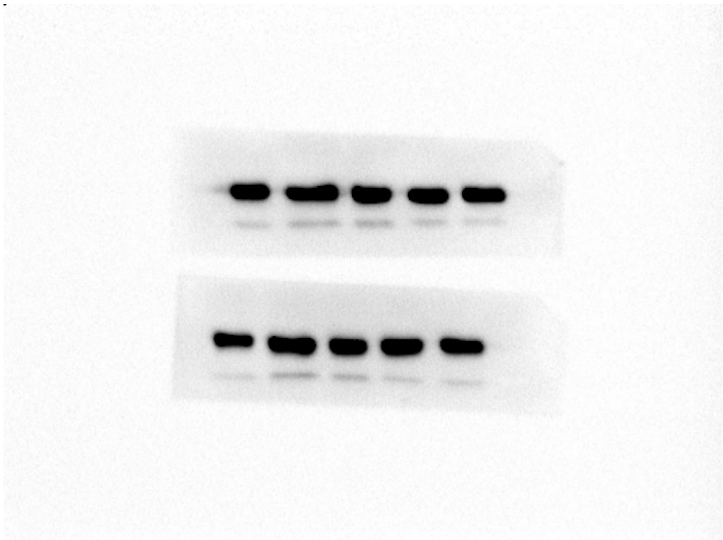

Supplementary Figure 8

cl-caspase3: 19kDa

The following blot: (The shallow one is the target protein): representative image

The sample oder: Sham, Control, SASP, Naringin(20mg/kg), Naringin(40mg/kg)

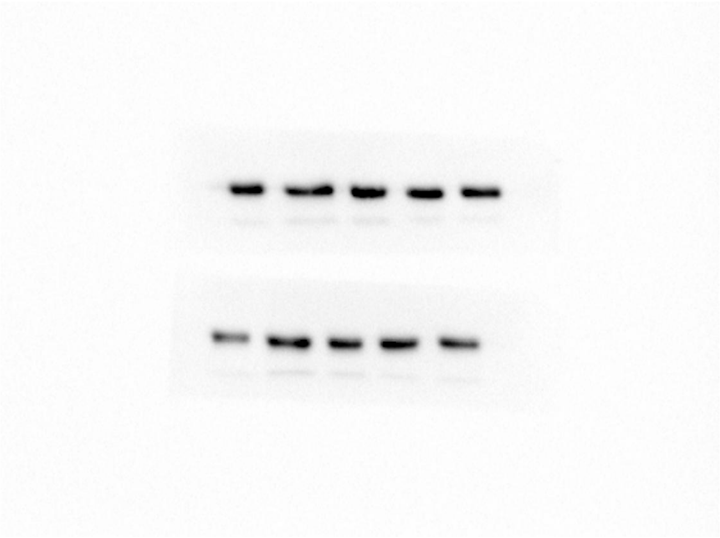

Supplementary Figure 9

GAPDH: 36kDa

The above blot: representative image

The sample oder: Sham, Control, SASP, Naringin(20mg/kg), Naringin(40mg/kg)

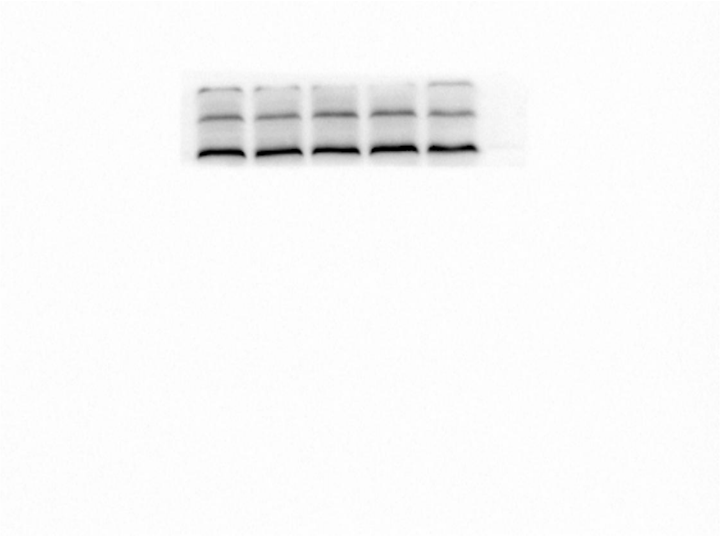

Supplementary Figure 10

PPAR-α: 54kDa

The following blot: representative image

The sample oder: Sham, Control, SASP, Naringin(20mg/kg), Naringin(40mg/kg)

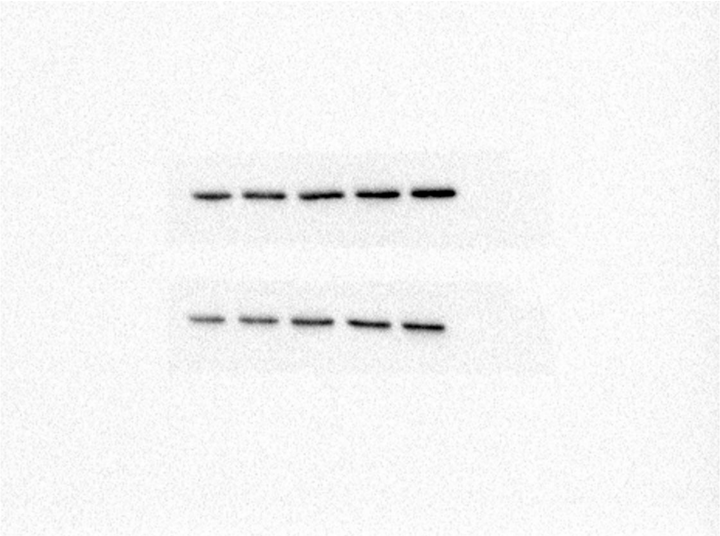

Supplementary Figure 11

GAPDH: 36kDa

The above blot: representative image

The sample oder: Sham, Control, SASP, Naringin(20mg/kg), Naringin(40mg/kg)

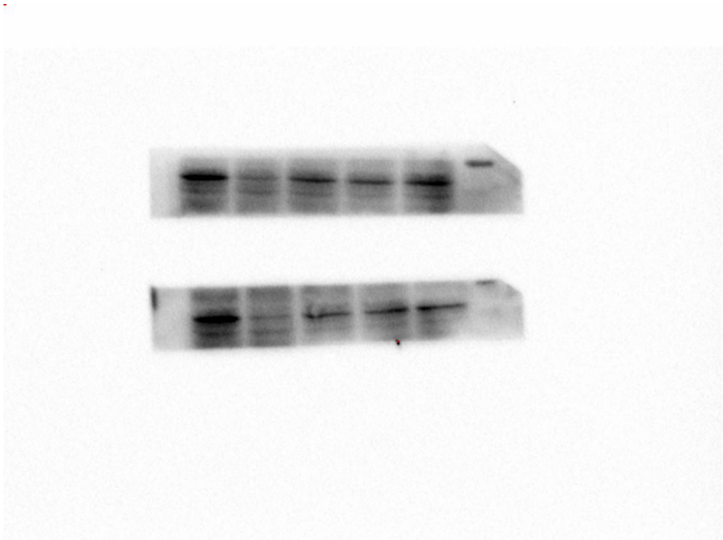

Supplementary Figure 12

PPAR-γ: 57kDa

The above blot: representative image

The sample oder: Sham, Control, SASP, Naringin(20mg/kg), Naringin(40mg/kg)

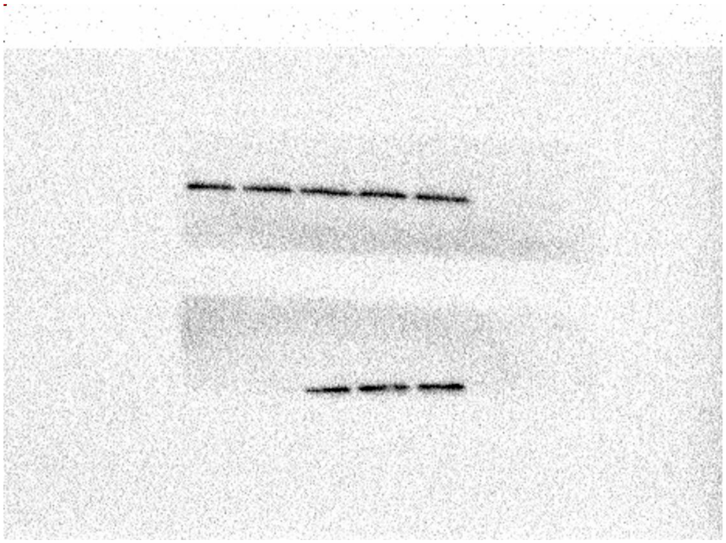

Supplementary Figure 13

GAPDH: 36kDa

The above blot: representative image

The sample oder: Sham, Control, SASP, Naringin(20mg/kg), Naringin(40mg/kg)

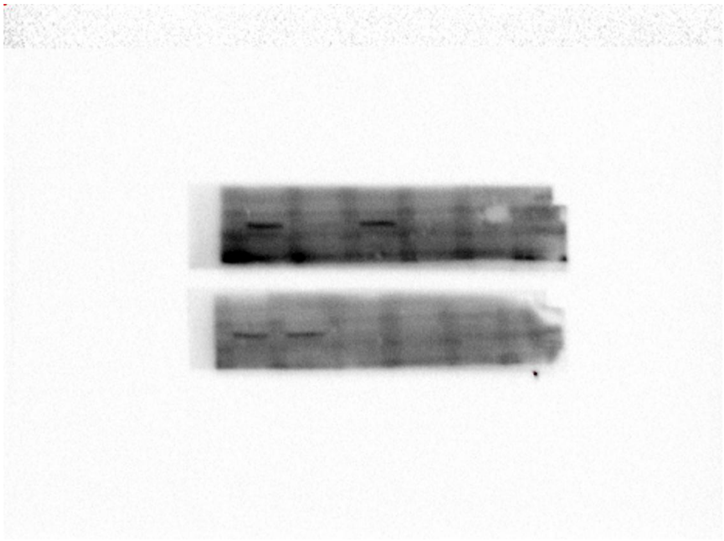

Supplementary Figure 14

PPAR-γ: 57kDa

The above blot: representative image

The sample oder: Sham, DSS, DSS+Naringin, DSS+BADGE, DSS+BADGE+Naringin

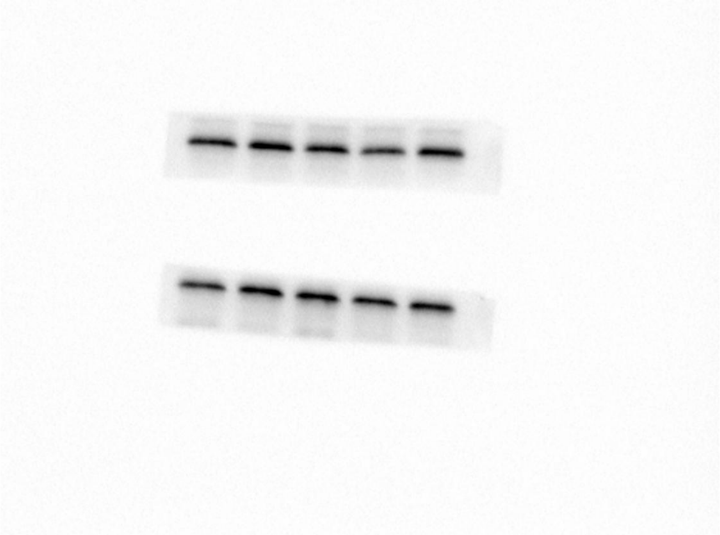

Supplementary Figure 15

GAPDH: 36kDa

The above blot: representative image

The sample oder: Sham, Control, SASP, Naringin(20mg/kg), Naringin(40mg/kg)

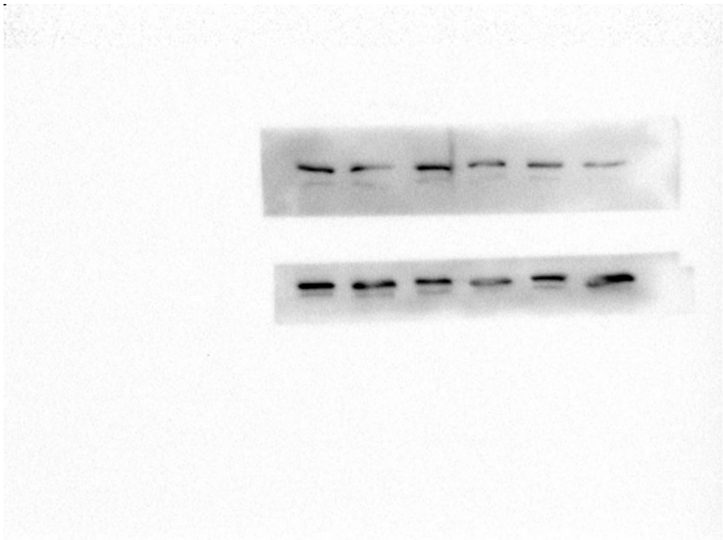

Supplementary Figure 16

PPAR-γ: 57kDa

The above blot: representative image

The sample oder: NC, LPS+siRNA-PPAR-γ control, LPS+siRNA-PPAR-γ control+Naringin, NC, LPS+siRNA-PPAR-γ, LPS+siRNA-PPAR-γ+Naringin

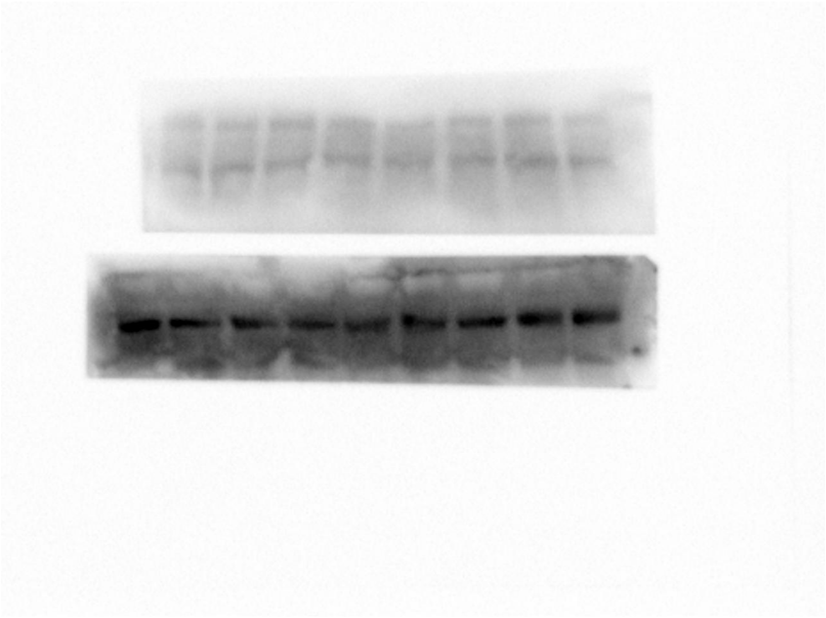

Supplementary Figure 17

p-NF-κB-p65: 65kDa

The following blot: representative image

The sample oder: Start with the fourth sample: NC, LPS+siRNA-PPAR-γ control, LPS+siRNA-PPAR-γ control+Naringin, NC, LPS+siRNA-PPAR-γ, LPS+siRNA-PPAR-γ+Naringin

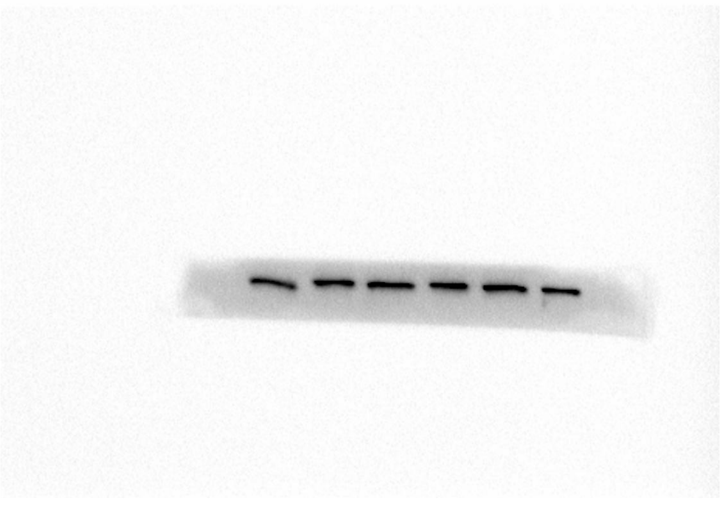

Supplementary Figure 18

GAPDH: 36kDa

The sample oder: NC, LPS+siRNA-PPAR-γ control, LPS+siRNA-PPAR-γ control+Naringin, NC, LPS+siRNA-PPAR-γ, LPS+siRNA-PPAR-γ+Naringin

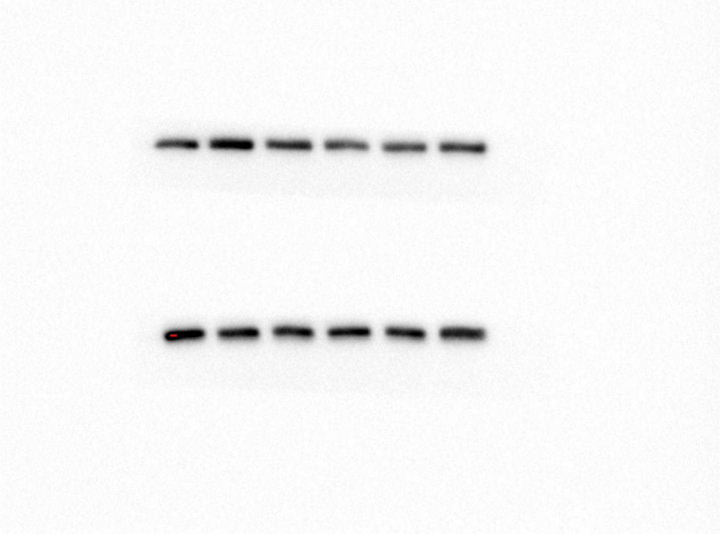

Supplementary Figure 19

PPAR-γ: 57kDa

The above blot: representative image

The sample oder: NC, LPS+siRNA-PPAR-γ control, LPS+siRNA-PPAR-γ control+Naringin, NC, LPS+siRNA-PPAR-γ, LPS+siRNA-PPAR-γ+Naringin

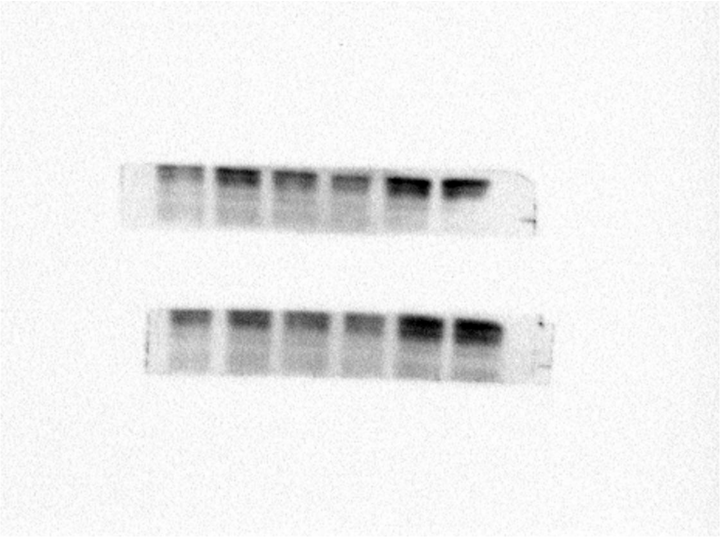

Supplementary Figure 20

p-NF-κB-p65: 65kDa

The above blot: representative image

The sample oder: NC, LPS+siRNA-PPAR-γ control, LPS+siRNA-PPAR-γ control+Naringin, NC, LPS+siRNA-PPAR-γ, LPS+siRNA-PPAR-γ+Naringin

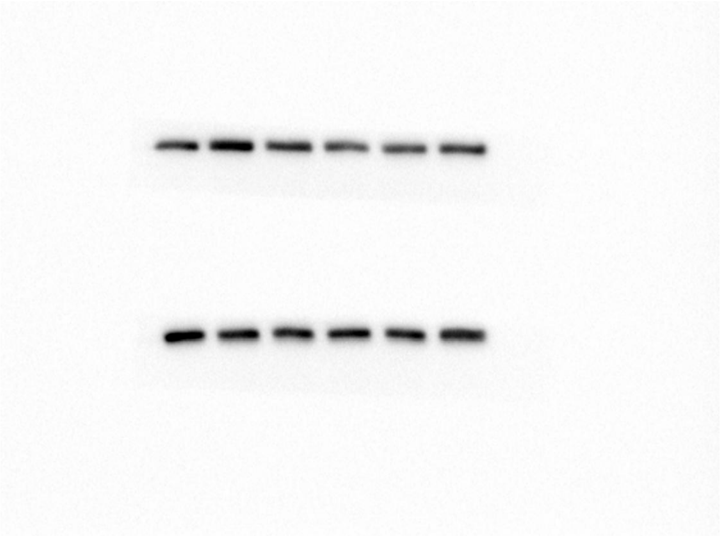

Supplementary Figure 21

GAPDH: 36kDa

The above blot: representative image

The sample oder: NC, LPS+siRNA-PPAR-γ control, LPS+siRNA-PPAR-γ control+Naringin, NC, LPS+siRNA-PPAR-γ, LPS+siRNA-PPAR-γ+Naringin

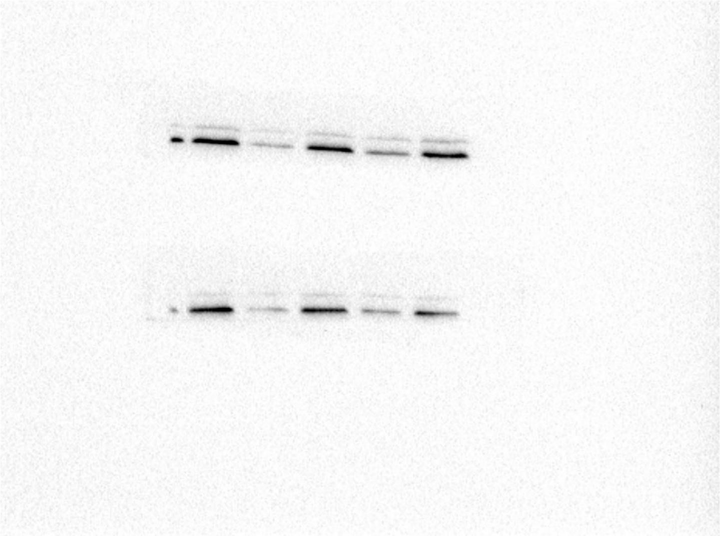

Supplementary Figure 22

PPAR-γ: 57kDa

The above blot: representative image

The sample oder: Sham, Control, SASP, Naringin(20mg/kg), Naringin(40mg/kg)

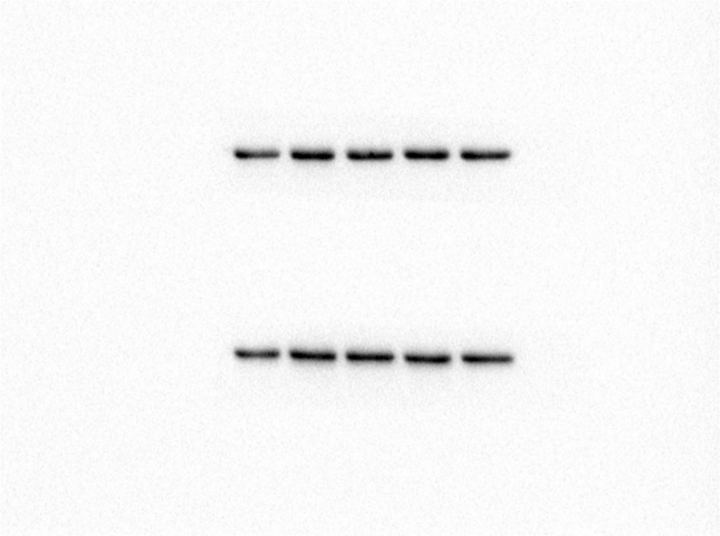

Supplementary Figure 23

GAPDH: 36kDa

The above blot: representative image

The sample oder: Sham, Control, SASP, Naringin(20mg/kg), Naringin(40mg/kg)

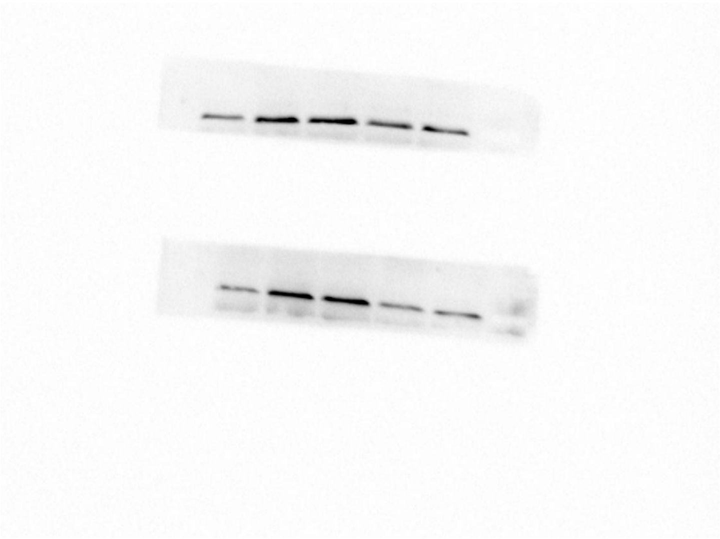

Supplementary Figure 24

p-NF-κB-p65: 65kDa

The following blot: representative image

The sample oder: Sham, Control, SASP, Naringin(20mg/kg), Naringin(40mg/kg)

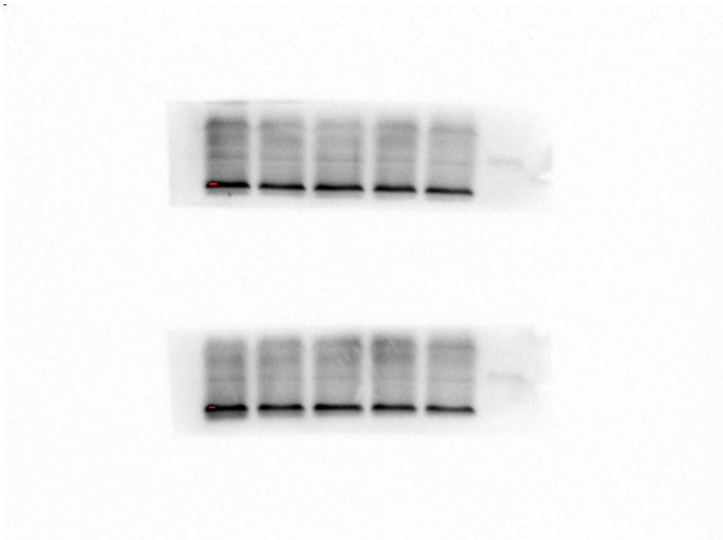

Supplementary Figure 25

NF-κB-p65: 65kDa

The above blot: representative image

The sample oder: Sham, Control, SASP, Naringin(20mg/kg), Naringin(40mg/kg)

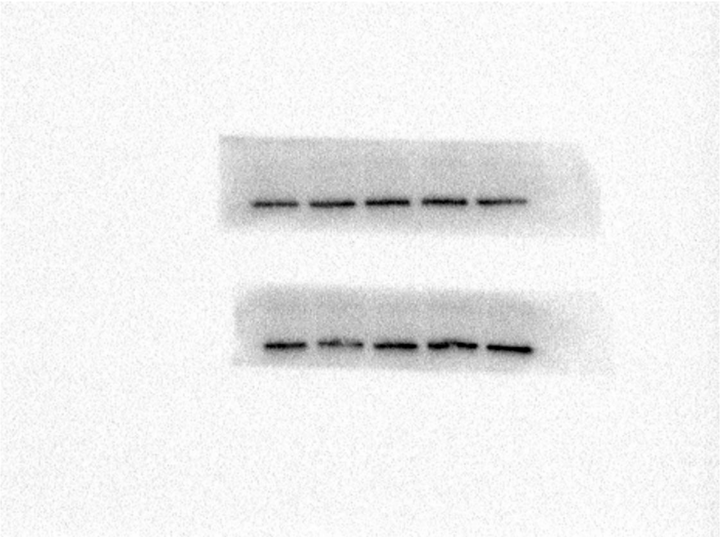

Supplementary Figure 26

GAPDH: 36kDa

The above blot: representative image

The sample oder: Sham, Control, SASP, Naringin(20mg/kg), Naringin(40mg/kg)

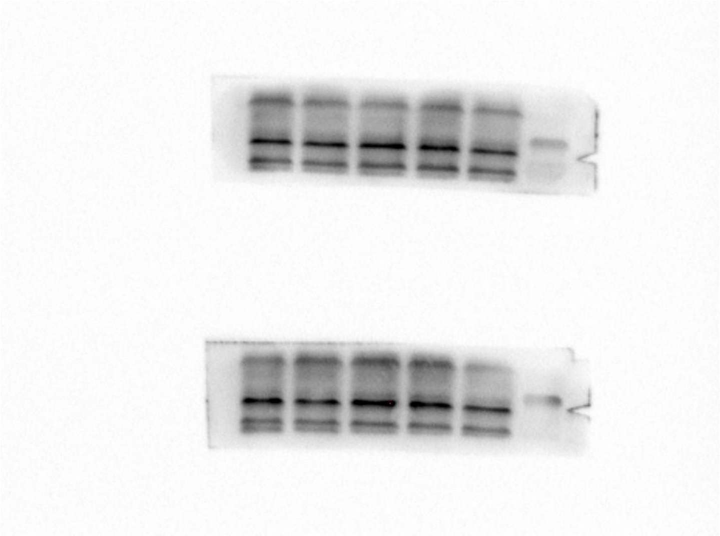

Supplementary Figure 27

iNOS: 131kDa

The above blot:representative image

The sample oder: Sham, Control, SASP, Naringin(20mg/kg), Naringin(40mg/kg)

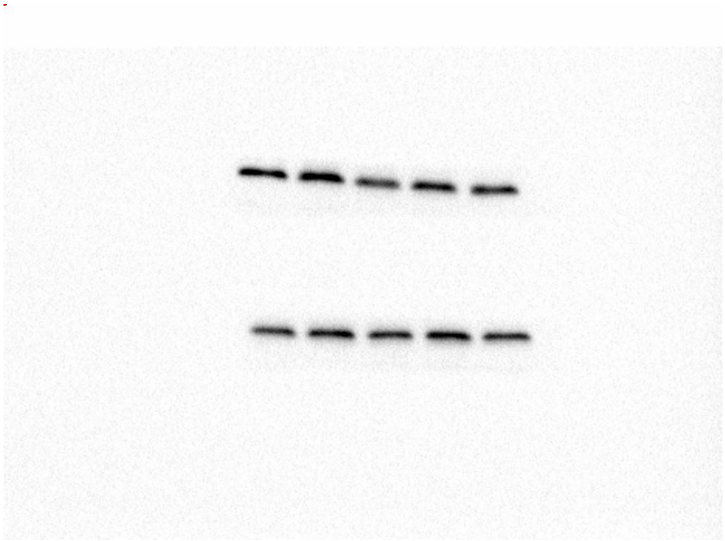

Supplementary Figure 28

GAPDH: 36kDa

The above blot: representative image

The sample oder: Sham, Control, SASP, Naringin(20mg/kg), Naringin(40mg/kg)

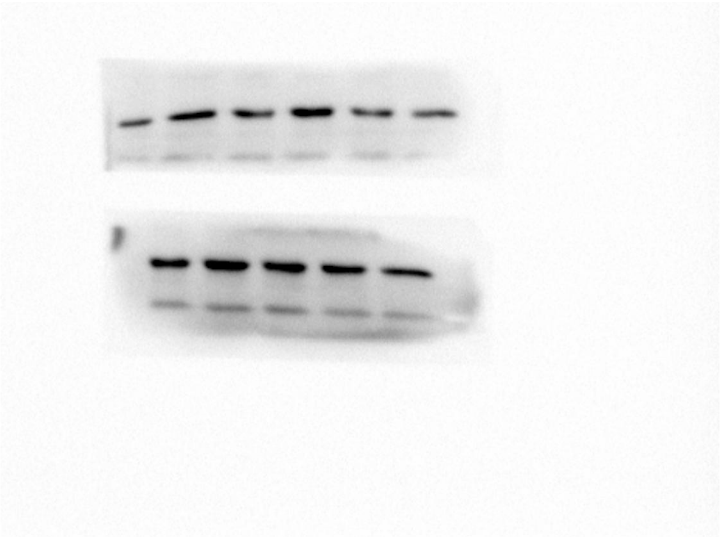

Supplementary Figure 29

caspase3: 32kDa

The following blot: representative image

The sample oder: Sham, Control, SASP, Naringin(20mg/kg), Naringin(40mg/kg)

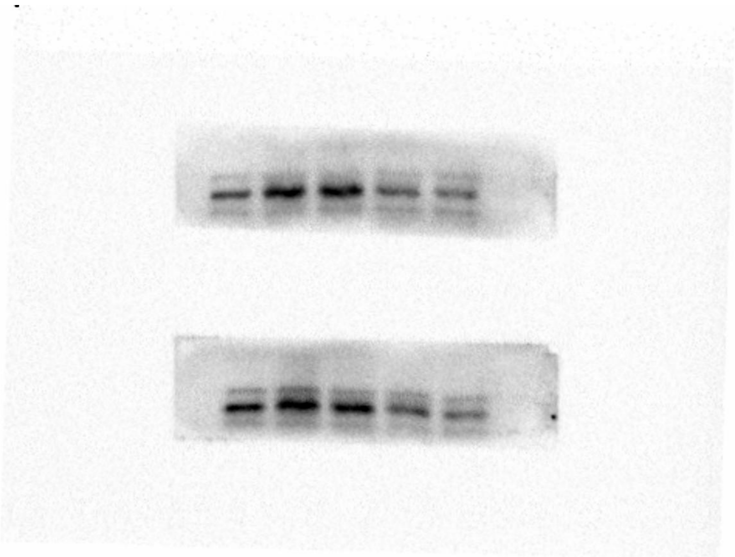

Supplementary Figure 30

cl-caspase3: 19kDa

The above blot: representative image

The sample oder: Sham, Control, SASP, Naringin(20mg/kg), Naringin(40mg/kg)

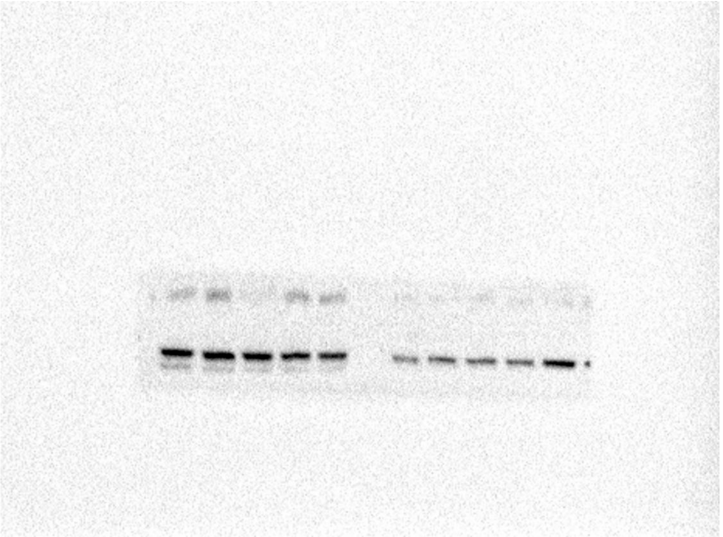

Supplementary Figure 31

GAPDH: 36kDa

The left blot: representative image

The sample oder: Sham, Control, SASP, Naringin(20mg/kg), Naringin(40mg/kg)
